# Changes in soil physicochemical properties and soil bacterial community in mulberry (*Morus alba* L.)/alfalfa (*Medicago sativa* L.) intercropping system

**DOI:** 10.1002/mbo3.555

**Published:** 2018-03-13

**Authors:** Meng‐Meng Zhang, Ning Wang, Yan‐Bo Hu, Guang‐Yu Sun

**Affiliations:** ^1^ College of Life Science Northeast Forestry University Harbin Heilongjiang China

**Keywords:** Alfalfa, Bacterial community, Intercropping, MiSeq sequencing, Mulberry

## Abstract

A better understanding of tree‐based intercropping effects on soil physicochemical properties and bacterial community has a potential contribution to improvement of agroforestry productivity and sustainability. In this study, we investigated the effects of mulberry/alfalfa intercropping on soil physicochemical properties and soil bacterial community by MiSeq sequencing of bacterial 16S rRNA gene. The results showed a significant increase in the contents of available nitrogen, available phosphate, available potassium, and total carbon in the rhizosphere soil of the intercropped alfalfa. Sequencing results showed that intercropping improved bacterial richness and diversity of mulberry and alfalfa based on richness estimates and diversity indices. The relative abundances of *Proteobacteria*,* Actinobacteria,* and *Firmicutes* were significantly higher in intercropping mulberry than in monoculture mulberry; and the abundances of *Proteobacteria*,* Bacteroidetes,* and *Gemmatimonadetes* in the intercropping alfalfa were markedly higher than that in monoculture alfalfa. Bacterial taxa with soil nutrients cycling were enriched in the intercropping system. There were higher relative abundances of *Bacillus* (0.32%), *Pseudomonas* (0.14%), and *Microbacterium* (0.07%) in intercropping mulberry soil, and *Bradyrhizobium* (1.0%), *Sphingomonas* (0.56%), *Pseudomonas* (0.18%), *Microbacterium* (0.15%), *Rhizobium* (0.09%), *Neorhizobium* (0.08%), *Rhodococcus* (0.06%), and *Burkholderia* (0.04%) in intercropping alfalfa soil. Variance partition analysis showed that planting pattern contributed 26.7% of the total variation of bacterial community, and soil environmental factors explained approximately 56.5% of the total variation. This result indicated that the soil environmental factors were more important than the planting pattern in shaping the bacterial community in the field soil. Overall, mulberry/alfalfa intercropping changed soil bacterial community, which was related to changes in soil total carbon, available phosphate, and available potassium.

## 1 INTRODUCTION

Soil plays a vital role in maintaining the balance of earth's ecosystem (Nesme, Colomb, Hinsinger, & Watson, 2014). Soil microorganisms, including protozoa, fungi, bacteria, and archaea, are an important component in soil; they play important roles in the biogeochemical cycle (Van Der Heijden, Bardgett, & Van Straalen, 2008), particularly in the processes of nutrient cycling, system stability, antijamming capability, and sustainable development of soil (Gyaneshwar, Kumar, Parekh, & Poole, 2002).

Intercropping has become a common practice in the world. Compared with a monoculture system, an intercropping system has obvious advantages in crop yield and stability (Willey, 1979), land‐use efficiency by enhancing light (Ghanbari, Dahmardeh, Siahsar, & Ramroudi, 2010), water (Morris & Garrity, 1993), nutrient use (Zhang & Li, 2003), and controlling on weeds (Liebman & Dyck, 1993), insects (Girma, Rao, & Sithanantham, 2000), or diseases (Zhang, Mallik, & Zeng, 2013). Land utilization is a major factor affecting soil microbiological indicators (Bissett, Richardson, Baker, & Thrall, 2011; Ding et al., 2013). Intercropping and crop rotation systems were reported to have positive impacts on soil microbial biomass and activity (Balota, Colozzi‐Filho, Andrade, & Dick, 2003) and populations of beneficial microorganisms such as the nitrogen‐fixing bacteria (Hungria & Vargas, 2000). Agroforestry, a tree‐based intercropping system, has a beneficial effect on biomass production, nutrients loss (Shanker & Solanki, 2000; Szott, Fernandes, & Sanchez, 1991), soil fertility through nitrogen fixation (Kass, Sylvester‐Bradley, & Nygren, 1997), organic matter production, and soil erosion.

Salinity and drought in northwest region of Heilongjiang Province in China limit agricultural and forest production. Mulberry (*Morus spp*.) is capable of moderate tolerance to salinity and drought, which has great economic importance as its leaves are highly palatable and digestible (70%–90%) to herbivorous animals (Vijayan, 2009). Mulberry leaves can also be fed to monophagous silkworm and monogastrics (Jetana, Vongpipatana, Thongruay, Usawang, & Sophon, 2010). Agroforestry system of intercropping between mulberry and crops has become an important planting pattern to bring economic development in this region. Recent studies showed that the application of tree intercropped with leguminous crops can be an especially sustainable and beneficial agricultural practice, as the N‐fixing crop provides natural N fertilizer for tree growth (Li, Sun, Zhang, Xu, & Sun, 2016; Peng, Zhang, Cai, Jiang, & Zhang, 2009; Rivest, Cogliastro, Bradley, & Olivier, 2010). Nitrogen‐fixing legumes may be improved by intercropping (Neumann, Schmidtke, & Rauber, 2007), because the intercropped trees can be more competitive for nitrogen in soil, forcing the legume crop to fix more atmospheric N_2_ (Hauggaard‐Nielsen, Ambus, & Jensen, 2003). In this study, the effects of the mulberry/alfalfa intercropping system on soil physicochemical properties and bacterial community of crop rhizosphere were analyzed. In the open field, mulberry and alfalfa were planted in intercropping and monoculture pattern. Soil bacterial community diversity, abundance, and composition were analyzed by MiSeq sequencing. We hypothesize that soil bacterial communities differ between mulberry and alfalfa as planting pattern conversion can induce changes in availability of plant‐derived nutrients. Plants have species‐specific effects on soil microbial communities (Bever, Platt, & Morton, 2012), and tree‐based intercropping systems support a more diverse soil microbial community compared to conventional agricultural systems (Bainard, Koch, Gordon, & Klironomos, 2013). Thus, we also hypothesize that soil bacterial community composition change and its diversity increase after intercropping.

## 2 MATERIALS AND METHODS

### 2.1 Site description and soil sampling

In 2011, the experimental field was established by the Institute of Crops, Heilongjiang Academy of Land Reclamation and Agricultural Sciences in Jiamusi city, Heilongjiang Province (46°46′N, 130°27′E), P.R. China. The treatments include the following: (1) monoculture mulberry (MM), (2) intercropping mulberry (IM), (3) monoculture alfalfa (MA), and (4) intercropping alfalfa (IA). The soil is a meadow soil with organic nitrogen 0.58 g·kg^−1^, available phosphorus 128.2 mg·kg^−1^, available potassium 106 mg·kg^−1^, and pH 6.8. The field site had been previously used for monoculture alfalfa. The samples for the study described here were taken in 2013, thus in the third year after establishment. The same soil preparations, row spacing, fertilization, and harvesting procedures were used in 3 years. Three plots (replications) of 5 m × 7.26 m were set up for each treatment and randomly distributed in the field. All treatments received farmyard manure at an application dose of 30,000 kg·hm^−2^ and ammonium phosphate of 150 kg·hm^−2^. The field management was carried out according to routine management.

The cultivars used were *Morus alba* “Qinglong” and *Medicago sativa* “Zhaodong”. Mulberry saplings with a height of 30 cm were cultivated in mid‐April (0.67 m interplant distance and 11 plants per row), and alfalfa seeds were sown in early April 2012 (The seeding amount is 22.5 kg·hm^−2^). In the intercropping system, two rows of mulberry trees and two rows of alfalfa (0.66 m inter‐row distance) were intercropped, with a total of 12 rows in each plot. The inter‐row spacing in monoculture was the same as in intercropping.

The mulberry and alfalfa rhizosphere soils were randomly sampled by the five‐point sampling method in each plot of the monoculture systems and from the central rows in each plot of the intercropping systems by uplifting intact roots on 16 August 2013 (Alfalfa generally reached early flowering stage). After shaking off the loosely adhered soils, the soils tightly adhering to root surface were brushed off and collected as the rhizosphere soil samples (Nazih, Finlay‐Moore, Hartel, & Fuhrmann, 2001). The rhizosphere soils obtained from the three plots were mixed and transported immediately to the laboratory. Each of the soil samples was sieved (<2 mm) and then divided into two aliquots in sealed bags: one aliquot was stored at −80°C for further use (DNA extraction), and the other aliquot was stored at 4°C for determination of soil physicochemical properties.

### 2.2 Soil physicochemical property

Soil available nitrogen was measured by alkaline hydrolysis diffusion method. For soil available phosphate and available potassium, the soils were first extracted with 0.5 mol/L sodium bicarbonate solution and 1 mol/L ammonium acetate solution, respectively, and then measured by Continuous Segmented Flow Analyzer (AutoAnalyzer 3, Seal Analytical, Germany). Total soil carbon was measured with chromic acid digestion by wet combustion.

### 2.3 Soil DNA extraction, PCR amplification, and MiSeq sequencing

DNA was extracted from the soil samples using PowerSoil DNA Isolation Kit (Mo Bio Laboratories, USA) according to the manufacturer's protocol and then quantified with 1% agarose gel electrophoresis. Extracted DNA from each sample was used as a template for amplification; the V4‐V5 hypervariable region of the 16S rRNA gene (Biddle, Fitz‐Gibbon, Schuster, Brenchley, & House, 2008) was amplified three times using the primer F515: GTGCCAGCMGCCGCGG, and primer R907: CCGTCAATTCMTTTRAGTTT, with unique barcode sequence at the 5′‐end of each primer, respectively. Each sample was amplified in triplicate with a 20‐μl reaction mixture containing 4 μl of 10 × Fast Pfu Buffer, 2 μl of 2.5 mmol/L dNTPs, 0.8 μl of each forward and reverse primers (5 mmol/L final concentration), 10 ng of template DNA, and 0.4 μl of Fast Pfu polymerase under the following conditions: an initial denaturation at 95°C for 2 min; 25 cycles of denaturation at 95°C for 30 s, annealing at 55°C for 30 s, and extension at 72°C for 1 min; with a final extension at 72°C for 10 min. The PCR products from same samples were pooled together and quantified by 2% agarose gel electrophoresis, then recycled and purified with AxyPrep DNA Purification Kit (AXYGEN Corporation), and quantified by 2% agarose gel electrophoresis and quantified with PicoGreen—Invitrogen by QuantiFluor™—ST (Promega Corporation). Finally, to dilute the PCR products of different samples based on sequencing request, a MiSeq library was constructed, followed by high‐throughput sequencing.

## 3 BIOINFORMATION ANALYSIS

### 3.1 Sequence optimization and data statistics/processing the sequencing data

MiSeq sequencing obtained the paired‐end (PE) reads. According to overlap relationship of PE reads at first, merging couples of reads to be a complete sequence, then the quality of reads and effects of merging were quality controlled and filtered; finally, valid sequences were obtained based on the barcodes of both ends of sequences and primer sequences differing samples, and direction of which was revised.

### 3.2 OTU cluster and taxonomy

Sequencing results of samples were defined as operational taxonomic units by bioinformation statistical analysis with a phylotype threshold of ≥97% sequence similarity. Software: Uesearch platform (version 7.1 http://drive5.com/uparse/). Based on the representative sequences of OTUs (≥97% similarity), species taxonomic information of each OUT can be obtained with Silva blast database (Quast et al., 2012) (Release 119 http://www.arb-silva.de).

### 3.3 Rarefaction curves, alpha diversity, and beta diversity

The trimmed sequences were conducted by the way of random sampling, in which rarefaction curves were established by the extracted sequences and numbers of these relevant representative OTUs.

Based upon OTU (≥97% similarity) data, alpha diversity was assessed calculating the richness estimators (The Chao1 estimator and ACE estimator show community richness), the diversity indices (the Shannon index and Simpson index show community diversity including richness and evenness), and coverage which shows sequencing depth.

Beta diversity using the biological distance of different samples according to species was calculated using the Bray–Curtis distance.

### 3.4 Statistical analysis

Significant differences between treatments were assessed by ANOVA, using Duncan's multiple range test. Data were subjected to two‐way ANOVA test using intercropping and crop type as sources of variable. ANOVA tests were performed using SPSS version 19. Based on OTU data, redundancy analysis was performed using Canoco for Windows 4.5, and Venn diagram, hierarchical cluster analysis, distance heatmap, redundancy analysis, and variation partition analysis were performed using R i386 3.2.3.

## 4 RESULTS

### 4.1 Physicochemical properties of rhizosphere soil of mulberry and alfalfa

Intercropping significantly affected the contents of available nitrogen, phosphate, potassium, and total carbon of crop rhizosphere soil (Table 1). The available nitrogen, phosphate, and potassium significantly decreased in the rhizosphere soil of intercropped mulberry, whereas they were significantly increased in the rhizosphere soil of intercropped alfalfa.

**Table 1:** Physicochemical parameters of different treatments

Sample	AN mg/kg	AP mg/kg	AK mg/kg	TC %
MM	148.9 ± 6.96ab	80.2 ± 0.38b	5.0 ± 0.70c	2.2 ± 0.00a
IM	123.1 ± 10.88a	52.53 ± 0.02a	3.4 ± 0.00b	2.2 ± 0.00a
MA	111.4 ± 3.37a	48.8 ± 5.31a	3.0 ± 0.07a	2.2 ± 0.00a
IA	227.3 ± 87.84b	80.0 ± 0.51b	6.3 ± 0.21d	2.5 ± 0.00b
Results of two‐way ANOVA test				
Int	2.73	1.29	144.90**	11.49**
Crt	3.23	182.40**	710.75**	9.00**

Values are means ± standard deviation (*n* = 3). Different lowercase letters meant significant differences among different samples according to the Duncan's multiple range test (*p *<* *.05).

AN, AP, AK, and TC represent available nitrogen, available phosphorus, available potassium, and total carbon. MM, IM, MA, and IA represent monocultured mulberry, intercropped mulberry, monocultured alfalfa, and intercropped alfalfa, respectively. Int and Crt represent intercropping treatment and crop type, respectively. **p <* *.01. The same abbreviations appear below.

### 4.2 Microbial richness and diversity

A total of 264,393 16S rRNA sequences were obtained from the eight soil samples by MiSeq analysis of the V3‐V4 region of bacterial 16S rRNA genes. The optimized sequence numbers for each sample ranged from 27,147 to 39,832. As the read numbers per sample were uneven, all samples were randomly reduced to the same size using MOTHUR basing on the smallest read number (27,147).

OTUs were identified at a genetic distance of 3%; then almost all curves reached saturation, indicating the survey effort covered almost the full extent of taxonomic diversity, as the curves leveled off with increasing number of reads sampled (Figure S1).

Among all the samples, a total of 10,711 OTUs were obtained, ranging from 5246 in monoculture soil to 5465 in intercropping soil (Table 2). The coverage of each sample was estimated to be up to 99%. The mean ACE richness estimator in alfalfa rhizosphere soil was higher than that of mulberry rhizosphere soil. Moreover, the same result was obtained with the Chao 1 richness estimator. The richness and diversity estimators (ACE, Chao1 and Shannon) of intercropping mulberry and alfalfa soils were higher than those of the corresponding monoculture soils; regardless of monoculture and intercropping, the values of alfalfa soil were greater than mulberry soils, while the Simpson estimators show the concordant conclusion. It is concluded from the above result that intercropping improved overall bacterial richness and diversity.

**Table 2:** Bacterial richness and diversity of monoculture mulberry, intercropping mulberry, monoculture alfalfa, and intercropping alfalfa

Sample	Cluster distance (0.03)					
	OTUs	ACE	Chao1	Coverage	Shannon	Simpson
MM‐1	1210	1299	1309	0.9939	5.74	0.0118
MM‐2	1267	1358	1360	0.9937	5.80	0.0114
IM‐1	1359	1510	1513	0.9914	5.98	0.0061
IM‐2	1311	1449	1498	0.9916	5.93	0.0068
MA‐1	1391	1511	1508	0.9924	6.07	0.0054
MA‐2	1378	1514	1535	0.9918	6.03	0.0059
IA‐1	1390	1512	1515	0.9924	6.12	0.0048
IA‐2	1405	1542	1556	0.9917	6.13	0.0047
Total	10711	–	–	–	–	–

The description of abbreviation names is shown in Table 1. MM‐1 and MM‐2 represent two repeats of MM, IM‐1 and IM‐2 represent two repeats of IM, MA‐1 and MA‐2 represent two repeats of MA, and IA‐1 and IA‐2 represent two repeats of IA. The same abbreviations appear below.

### 4.3 Taxonomic composition of bacterial communities

**Figure 1:**
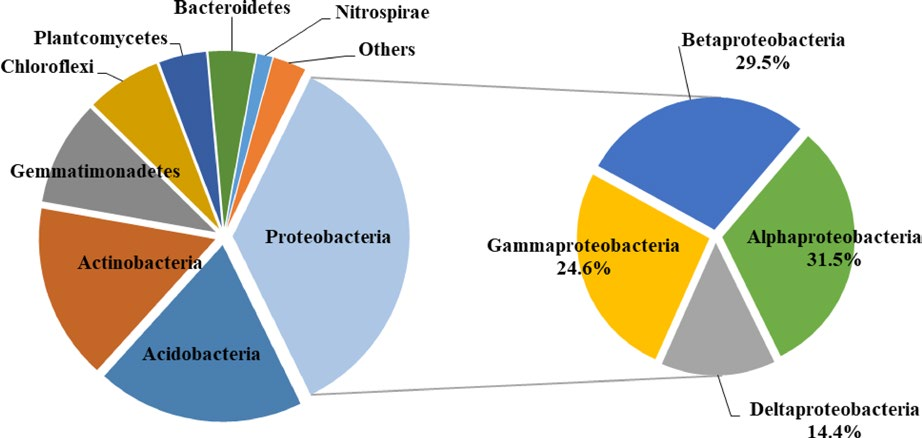
Relative abundance of soil bacterial phyla (left) and proteobacterial classes (right) in different treatments

All valid sequences from the soil sample libraries were classified from phylum to species. The results showed the differences of bacterial community abundance at different phylogenetic levels (Tables S1‐S3). Twenty‐eight phyla were found in all samples, among them, the dominant bacterial phyla were *Proteobacteria, Acidobacteria*,* Actinobacteria*,* Gemmatimonadetes, Chloroflexi, Planctomycetes, Bacteroidetes*, and *Nitrospirae*; they accounted for over 95% of the reads in each sample, representing 35.5%, 19.0%, 16.1%, 9.6%, 6.8%, 4.3%, 4.3%, and 1.4%, respectively (Figure 1left). The other sequences belonged to *Verrucomicrobia*,* Armatimonadetes*,* Firmicutes*,* Latescibacteria,* and the other 16 bacterial phyla; having very low proportions (<1%). The primary bacteria (*Proteobacteria*) mainly consisted of *alpha‐proteobacteria, beta‐proteobacteria*,* gamma‐proteobacteria,* and *delta‐proteobacteri*a (Figure 1 right).

**Figure 2:**
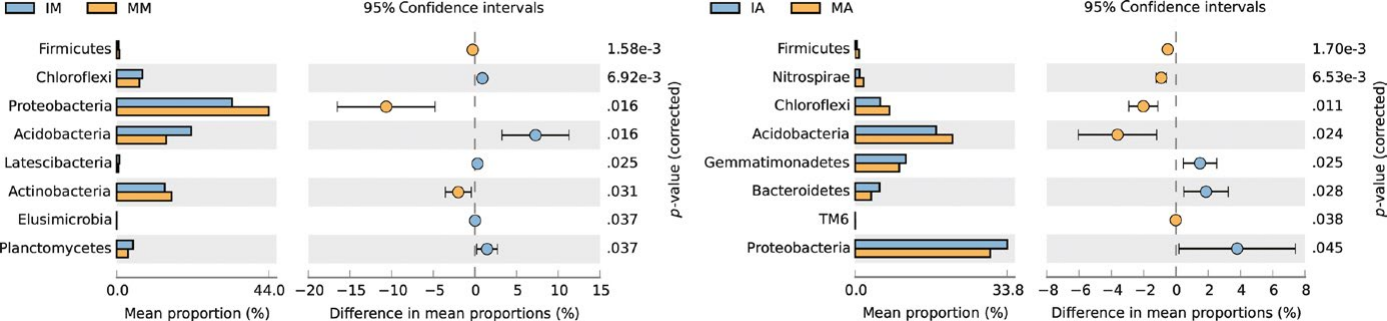
Abundance of bacterial phyla in monoculture and intercropping mulberry soil (left) and abundance of bacterial phyla in monoculture and intercropping alfalfa soil (right). Note: The figures only show bacterial phyla with relative abundance significantly different under different treatments. MM, IM, MA, and IA represent monocultured mulberry, intercropped mulberry, monocultured alfalfa, and intercropped alfalfa, respectively. MM‐1 and MM‐2 represent two repeats of MM, IM‐1 and IM‐2 represent two repeats of IM, MA‐1 and MA‐2 represent two repeats of MA, and IA‐1 and IA‐2 represent two repeats of IA. The same abbreviations appear below

In mulberry soil, *Proteobacteria*,* Actinobacteria,* and *Firmicutes* showed a higher abundance in intercropping treatment (*p *<* *.05), whereas *Acidobacteria*,* Chloroflexi*,* Planctomycetes*,* Latescibacteria,* and *Elusimicrobia* showed a higher abundance in monoculture treatment (*p *<* *.05) (Figure 2). In alfalfa soil, *Proteobacteria*,* Bacteroidetes,* and *Gemmatimonadetes* presented the dominant phyla in the intercropping treatment; *Acidobacteria*,* Chloroflexi*,* Nitrospirae,* and *Firmicutes* presented disadvantages in intercropping treatment.

**Figure 3:**
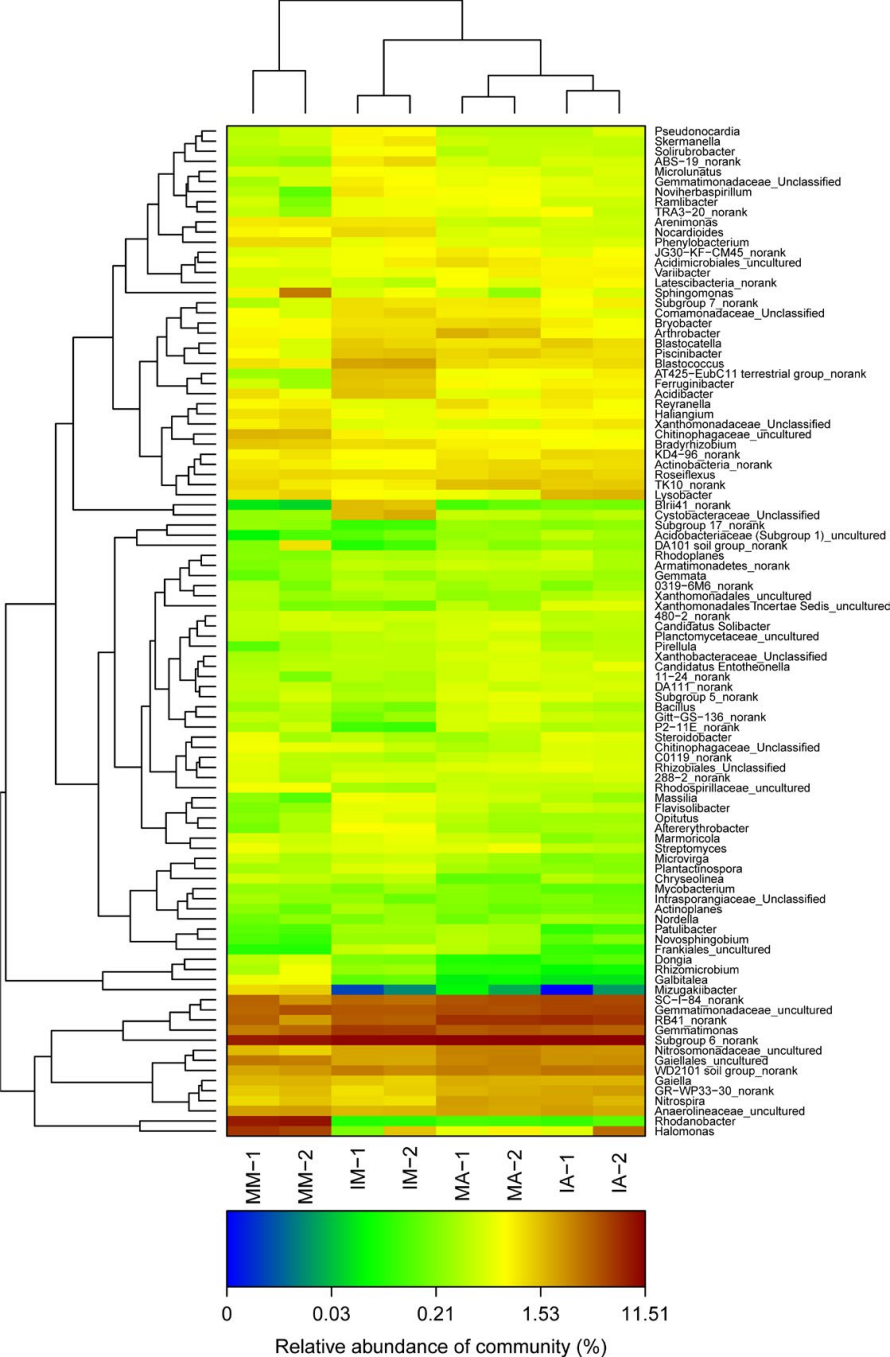
Hierarchical cluster analysis of 100 predominant bacterial communities in different treatments. The OTUs were ordered by genus. Samples communities were clustered based on complete linkage method. The color intensity of scale indicates relative abundance of each OTU read. Relative abundance was defined as the number of sequences affiliated with the OUT divided by the total number of sequences per sample

*Gemmatimonas*,* Halomonas*,* Gaiella*,* Nitrospira,* and *Roseiflexus* were the most abundant genera across all soil samples (Figure 3 and Table S3), representing 4.42%, 1.31%, 1.35%, 1.30%, and 1.06% of all classified sequences in the intercropping soils and 3.46%, 2.89%, 1.52%, 1.51%, and 1.01% in the monoculture soils, respectively. It indicated that these genera might be indigenous genus to the soil sampled. The distribution of related genus which involved in soil nutrient cycling varied between monoculture and intercropping soils. In the mulberry soils, *Bacillus* (0.32%), *Pseudomonas* (0.14%), and *Microbacterium* (0.07%) showed more relative abundances in intercropping, while *Sphingomonas* (1.74%), *Bradyrhizobium* (1.16%), *Arthrobacter* (0.74%), *Mesorhizobium* (0.22%), *Rhizobium* (0.10%), *Burkholderia* (0.05%), and *Neorhizobium* (0.02%) showed an opposite result. In the alfalfa soils, there were higher relative abundances of *Bradyrhizobium* (1.0%), *Sphingomonas* (0.56%), *Pseudomonas* (0.18%), *Microbacterium* (0.15%), *Rhizobium* (0.09%), *Neorhizobium* (0.08%), *Rhodococcus* (0.06%), and *Burkholderia* (0.04%) in intercropping, whereas *Arthrobacter* (1.47%), *Bacillus* (0.49%), and *Mesorhizobium* (0.12%) were more abundant in monoculture.

### 4.4 Shared bacterial OTUs

**Figure 4:**
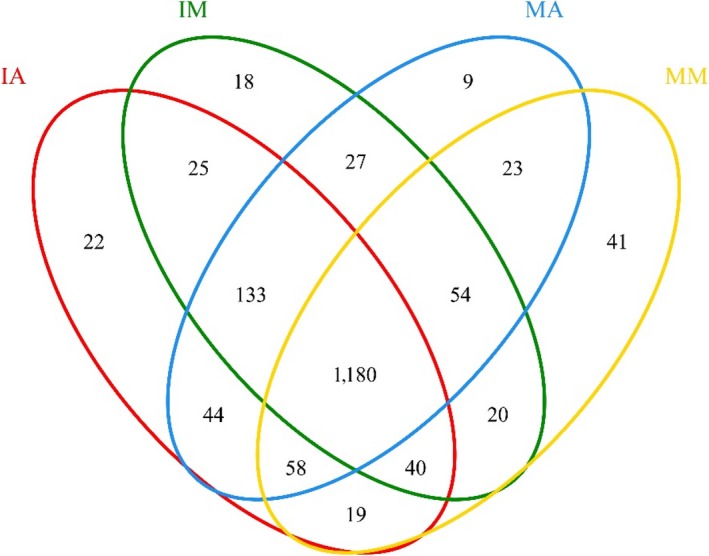
Venn diagram showing the shared bacterial OTUs in all soil samples

Venn diagrams revealed the total observed OTUs in soil samples (Figure 4), and 1,180 OTUs were common to all soil samples. Moreover, the distribution of sequences also demonstrated that each plant rhizosphere had its own microbial population.

**Figure 5:**
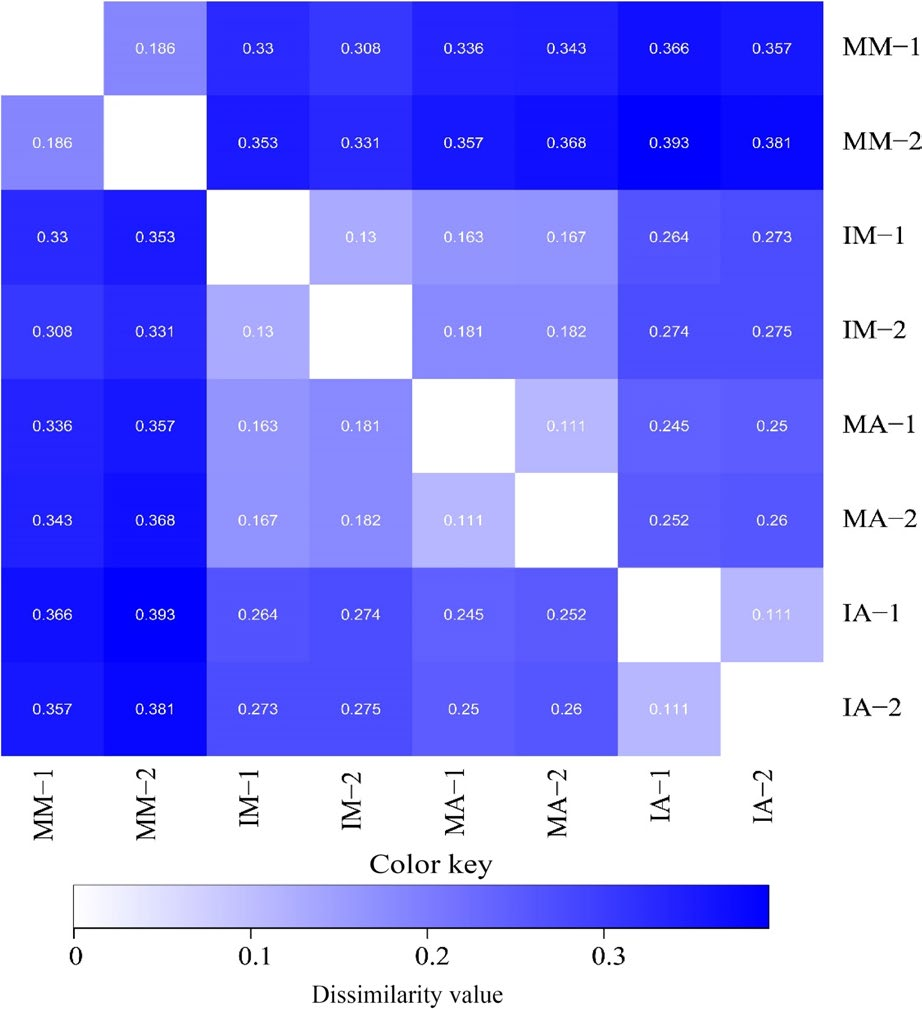
Distance Heatmap of a Bray–Curtis dissimilarity matrix in different treatments

Hierarchically clustered heatmap analysis, based on the microbial community profiles at the genus level, was used to identify the different compositions of the microbial community (Figure 3). The MM and IM groups were separated from MA and IA groups, suggesting the clear distinction of microbial community composition between mulberry and alfalfa groups. The dissimilarity matrix also showed big dissimilarity values among intercropping groups (IM and IA) and monoculture groups (MM and MA), respectively (Figure 5).

### 4.5 Correlations of soil properties, the dominant bacterial phyla, and bacterial communities

Soil available phosphate, available potassium, and total carbon were closely correlated with the abundance of the dominant bacterial phyla (Table 3). The abundance of *Acidobacteria* (*r* = −.89, *p *<* *.001), *Chloroflexi* (*r* = −.88, *p *<* *.001), *Planctomycetes* (*r* = −.81, *p *=* *.002), and *Nitrospirae* (*r* = −.93, *p *<* *.001) were significantly negatively correlated with soil available phosphate, while *Bacteroidetes* (*r* = .72, *p *=* *.008) was positively correlated with soil available phosphate. The abundance of *Chloroflexi* had a markedly negatively relationship with available potassium (*r* = −.99, *p *<* *.001) and total carbon (*r* = −.82, *p *=* *.001), and the *Bacteroidetes* exhibited a highly significant positive correlation with available potassium (*r* = .90, *p *<* *.001) and total carbon (*r* = .81, *p *=* *.001), then *Nitrospirae* (*r* = −.95, *p *<* *.001) were significantly negatively correlated with soil available potassium. Additionally, the soil available nitrogen has no significant correlations with the abundance of all the dominant bacterial phyla.

**Table 3:** Pearson's correlation (*r*) and significance (*p*) values between the abundances of the dominant abundant bacterial phyla and the soil variables

Taxonomic group	TC		AN		AP		AK	
	*r*	*p*	*r*	*p*	*r*	*p*	*r*	*p*
*Proteobacteria*	−.054	.867	.079	.807	.694	.012	.428	.165
*Acidobacteria*	−.248	.437	−.286	.367	−**.89**	<.001	−.69	.013
*Actinobacteria*	.684	.014	.437	.155	.432	.161	.53	.076
*Gemmatimonadetes*	.473	.121	.356	.256	−.2	.534	.141	.662
*Chloroflexi*	−**.822**	.001	−.626	.03	−**.881**	<.001	−**.991**	<.001
*Planctomycetes*	−.124	.701	−.279	.379	−**.806**	.002	−.585	.046
*Bacteroidetes*	**.814**	.001	.604	.038	**.724**	.008	**.903**	<.001
*Nitrospirae*	−.680	.015	−.627	.029	−**.934**	<.001	−**.951**	<.001

Values in bold indicate significant correlations (*p *<* *.01).

**Figure 6:**
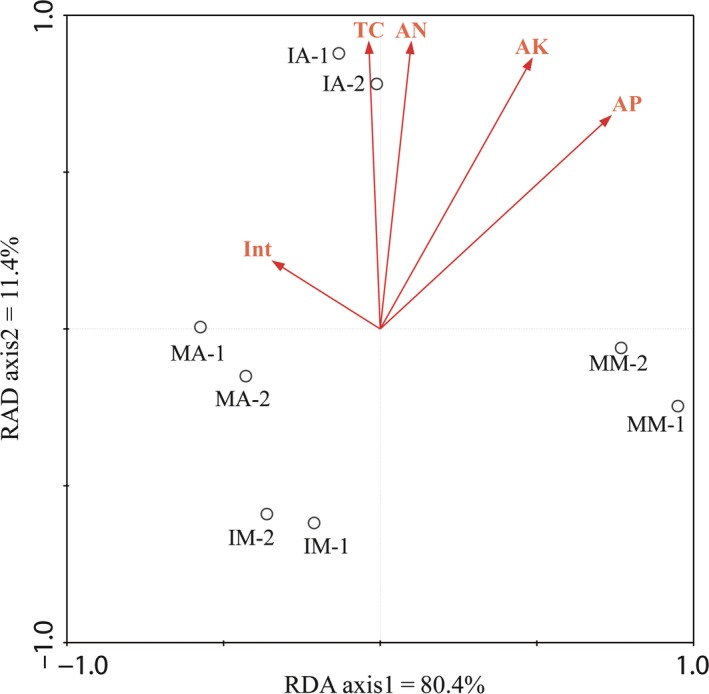
Redundancy analysis (RDA) for soil bacterial community, soil variables, and intercropping. Arrows indicated the direction and magnitude of measurable variables associated with bacterial community structures. Each circle represents a sample. Note:RDA component 1 and 2 explained 80.4% and 11.4% of the total variations, respectively. Just based on RDA1, eight samples were divided into two groups, MA and IA were clustered together, and IM was in the same group

To investigate relationships between soil bacterial community composition and soil variables, the OTUs from all soil samples were analyzed using Redundancy analysis (RDA) (Figure 6). Overall, the two RDA axes explained 91.8% of the variation between the soil bacterial communities. The distinctions of bacterial community structure among mulberry and alfalfa groups were also supported by the redundancy analysis (RDA). Alfalfa samples (MA and IA) were clustered together and were well separated from that mulberry samples (MM), and there was a big distinction between MM and IM samples, and an approaching trend between IM and MA. The results suggested that plant species had a great impact on the bacterial communities, while planting pattern also changed the bacterial community structure. Available phosphate and available potassium had the longest arrow, indicating that they were the most important factors affecting the bacterial community; total carbon and available nitrogen were secondary ones, and the planting pattern was the last one.

### 4.6 Bacterial community links to soil properties and planting pattern

**Figure 7:**
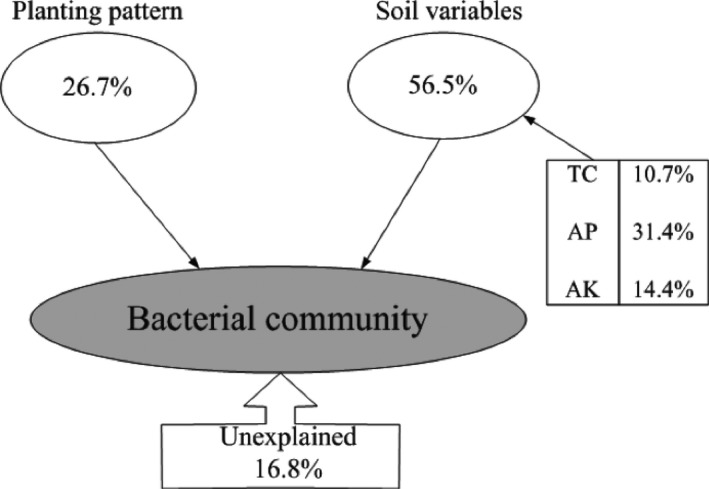
Variation partition analysis of the effects of planting pattern and soil variables on the phylogenetic structure of bacterial communities

Variance partitioning analysis showed that the combination of the selected soil variables and planting pattern showed a significant (*r* = .432, *p* = .001) correlation with the bacterial community structure. These parameters explained 83.2% of the bacterial community variation, leaving 16.8% of unexplained variation. The planting pattern explained 26.7% of the bacterial community variation. Among the selected soil parameters, soil total carbon, available phosphate, and available potassium explained 10.7%, 31.4%, and 14.4% of the bacterial community variation, respectively (Figure 7).

## 5 DISCUSSION

### 5.1 Soil physicochemical properties

In alfalfa soils, intercropping treatment significantly increased the contents of soil available nitrogen, phosphate, and potassium. Therefore, the intercropping system was beneficial to the availability of soil nutrients. A possible reason may be due to the increased abundances of related bacterium involved in soil nutrient cycling, for example, rhizobia, phosphate‐solubilizing, and potassium‐solubilizing bacteria; another reason may be the nutrients released by agroforestry trees to meet crops demands (Palm, 1995). Moreover, the intercropping alfalfa significantly increased soil total carbon content. However, soil available nitrogen, phosphate, and potassium contents decreased in the intercropping mulberry soil. Therefore, intercropping may affect soil physicochemical properties indirectly through plant‐mediated changes in soil microbial community.

### 5.2 Bacterial community diversity and structure

The analysis on richness estimators (Ace and Chao 1) and the diversity indices (Shannon and Simpson) revealed a richer bacterial community in intercropping soils than that of monoculture soils. Intercropping pepper with green garlic improved soil microbial properties as compared to monoculture (Ahmad et al., 2013). The tree‐based intercropping system presented a more heterogeneous vegetation cover and rooting pattern, thereby increasing the diversity of soil microbial communities (Lacombe, Bradley, Hamel, & Beaulieu, 2009).

In all samples, the dominant taxonomic groups were *Proteobacteria, Acidobacteria*,* Actinobacteria*,* Gemmatimonadetes, Chloroflexi, Planctomycetes, Bacteroidetes*, and *Nitrospirae* (Figure 1, left). These phyla have been depicted as common inhabitants of soil (Caporaso et al., 2012; Fierer et al., 2012). The *Proteobacteria* subgroups almost contributed to the entire *Proteobacteria* group (Figure 1, right). Several studies suggested that the interaction among plant species richness, soil types (despite management and seasonal variations), and land‐use history could affect microbial community structure (Chung, Zak, Reich, & Ellsworth, 2007; Drenovsky, Vo, Graham, & Scow, 2004; Jangid et al., 2011). The bacterial community structure in mulberry or alfalfa soils were different between the monoculture and intercropping system. The relative abundances of the dominant bacterial phyla (*Acidobacteria*,* Gemmatimonadetes,* and *Bacteroidetes*) were greater in intercropping systems than that in monoculture systems; the relative abundances of the dominant bacterial phyla (*Proteobacteria* and *Chloroflexi*) were greater in mulberry soils than that in alfalfa soils. These results indicated that both planting pattern and plant species changed the dominant bacterial phyla.

Nitrogen, phosphorus, and potassium are essential macronutrients that play an important role in the growth and development of plants ((Franche, Lindström, & Elmerich, 2009; Keshavarz Zarjani, Aliasgharzad, Oustan, Emadi, & Ahmadi, 2013; Sharma, Sayyed, Trivedi, & Gobi, 2013). In the intercropping system, the changes of bacterial taxa with the processes of nitrogen fixation, phosphate, and potassium solubilization were very important for plant growth and soil quality. The nitrogen fixation bacteria included *Rhizobium*,* Azotobacter*, and *Azospirillum* (Kumar & Rao, 2012). In this study, we found the other nitrogen fixation rhizobia such as *Bradyrhizobium, Mesorhizobium,* and *Neorhizobium*. Intercropping increased relative abundances of *Rhizobium*,* Neorhizobium,* and *Bradyrhizobium* in alfalfa soil. Previous studies showed a common result that nitrogen fixation capacity of legumes may be enhanced by intercropping when the nonlegume is a strong competitor for soil inorganic nitrogen (Giller, Ormesher, & Awah, 1991; Hauggaard‐Nielsen, Ambus, & Jensen, 2001; Karpenstein‐Machan & Stuelpnagel, 2000). Several phosphate‐solubilizing bacteria like *Pseudomonas*,* Bacillus*,* Rhizobium*,* Rhodococcus*,* Arthrobacter*,* Burkholderia,* and *Sphingomonas* have been considered as the best eco‐friendly means for mobilizing phosphorus nutrition for crops (Chen et al., 2006; Panhwar et al., 2014; Rodríguez & Fraga, 1999; Sharma et al., 2013). The phosphate‐solubilizing bacteria were present in different proportions in the monoculture and intercropping soils. Intercropping increased the relative abundance of *Pseudomona*s. *Bacillus* was more abundant in the soil of intercropping mulberry, while *Rhizobium*,* Rhodococcus*,* Burkholderia,* and *Sphingomonas* were more abundant in the soil of intercropping alfalfa. Several bacteria (*Bacillus*,* Microbacterium,* and *Burkholderia*) have been identified as having the ability to solubilize potassium (Basak & Biswas, 2009; Keshavarz Zarjani et al., 2013; Sheng & He, 2006; Sugumaran & Janarthanam, 2007; Zhang & Kong, 2014). *Microbacterium* showed more abundance in intercropping soils than in monoculture soils. These results demonstrated that soil bacterial community structure and diversity differed between intercropping and monoculture.

### 5.3 Effects of soil properties and planting pattern on the dominant bacterial phyla and bacterial community structure

To investigate the relationships between soil microbial community structure and measured soil variables in the monoculture and intercropping systems, we analyzed the dominant bacterial phyla and OTUs data using Pearson's correlation and RDA. Total carbon content had a positive correlation with the relative abundance of *Bacteroidetes* (*r* = .814, *p* = .001) and an negative correlation with the relative abundance of *Chloroflexi* (*r* = −.822, *p* = .001), which in accordance with previous observations that net carbon mineralization rate (an index of carbon availability) was a strong predictor of the abundances of three groups: *Acidobacteria* (a negative correlation), β*‐Proteobacteria* (a positive correlation), and *Bacteroidetes* (a positive correlation) (Fierer, Bradford, & Jackson, 2007). An accumulation of soil nutrients, especially carbon and soil organic matter, which accompanies succession, could directly affect soil microbial communities (Hooper et al., 2000; Liu et al., 2014). In the study, total soil carbon, available phosphate, and available potassium had positive or/and negative correlations with the dominant bacterial phyla; thus, our results support the conclusion that the soil variables have substantial impacts on the dominant bacterial phyla.

Redundancy analysis (RDA) showed that the monoculture alfalfa and intercropping mulberry were separated from the monoculture mulberry through the RDA component 1, and the monoculture alfalfa was separated from the intercropping alfalfa by the RDA component 2. The distance dissimilarity matrix also suggested that soil bacterial community differed among different plant species and planting patterns. The arrow length of available phosphate, available potassium, and total carbon indicated that they affected bacterial community structure. Variation partition analysis clearly quantized the effects of planting pattern and soil variables on soil bacterial community structure.

## 6 CONCLUSIONS

Our results demonstrated that intercropping improved the richness and diversity of bacterial communities and changed the structure of bacterial communities of both mulberry and alfalfa soils, which were linked to changes in soil total carbon, available phosphate, and available potassium content. Intercropping had positive impacts on soil quality by changing soil physicochemical properties and promoting soil beneficial bacterium participating soil nutrients cycling.

## CONFLICT OF INTERESTS

The authors have declared that no conflict of interests exists.
