# Supplementary material for: Changes in soil physicochemical properties and soil bacterial community in mulberry (Morus alba L.)/alfalfa (Medicago sativa L.) intercropping system
Source: Microbiologyopen. 2018 Mar 13;7(2):e00555. doi: 10.1002/mbo3.555 (PMC5912001; doi:10.1002/mbo3.555)
Supplement: Supplementary file 1 [file MBO3-7-na-s001.docx]

Table S1 Relative abundances of bacterial phylum in the studied soils.

Note：Values represent percentages of all sequences assigned to the bacteria for soils. MM-1 and MM-2 represent two repeats of MM, IM-1 and IM-2 represent two repeats of IM, MA-1 and MA-2 represent two repeats of MA, and IA-1 and IA-2 represent two repeats of IA. The same abbreviations appear below.

| **Phylum** | **MM-1** | **MM-2** | **IM-1** | **IM-2** | **MA-1** | **MA-2** | **IA-1** | **IA-2** |
| --- | --- | --- | --- | --- | --- | --- | --- | --- |
| *Acidobacteria* | 14.92 | 13.81 | 22.48 | 20.91 | 21.20 | 22.22 | 18.40 | 17.82 |
| *Actinobacteria* | 16.25 | 15.68 | 13.70 | 14.19 | 17.12 | 16.62 | 17.47 | 17.70 |
| *Aerophobetes* | 0.00 | 0.00 | 0.00 | 0.01 | 0.00 | 0.00 | 0.00 | 0.00 |
| *Armatimonadetes* | 0.42 | 0.50 | 0.74 | 0.61 | 0.81 | 0.77 | 0.69 | 0.71 |
| *Bacteria_Unclassified* | 0.09 | 0.07 | 0.17 | 0.14 | 0.15 | 0.14 | 0.12 | 0.11 |
| *Bacteroidetes* | 4.27 | 4.02 | 4.22 | 3.76 | 3.75 | 3.40 | 5.73 | 5.17 |
| *Caldiserica* | 0.00 | 0.02 | 0.00 | 0.00 | 0.00 | 0.00 | 0.00 | 0.00 |
| *Chlamydiae* | 0.00 | 0.01 | 0.00 | 0.00 | 0.00 | 0.00 | 0.00 | 0.00 |
| *Chlorobi* | 0.07 | 0.07 | 0.09 | 0.10 | 0.13 | 0.07 | 0.14 | 0.18 |
| *Chloroflexi* | 6.58 | 6.60 | 7.57 | 7.42 | 7.72 | 7.58 | 5.43 | 5.80 |
| *Cyanobacteria* | 0.04 | 0.03 | 0.01 | 0.01 | 0.08 | 0.04 | 0.14 | 0.13 |
| *Deinococcus-Thermus* | 0.00 | 0.00 | 0.00 | 0.00 | 0.00 | 0.00 | 0.00 | 0.01 |
| *Elusimicrobia* | 0.01 | 0.01 | 0.04 | 0.03 | 0.02 | 0.07 | 0.07 | 0.06 |
| *Firmicutes* | 0.79 | 0.78 | 0.53 | 0.52 | 0.87 | 0.90 | 0.38 | 0.37 |
| *Gemmatimonadetes* | 6.58 | 8.05 | 9.96 | 9.92 | 9.64 | 10.03 | 11.49 | 11.18 |
| *Hydrogenedentes* | 0.03 | 0.00 | 0.03 | 0.02 | 0.03 | 0.02 | 0.01 | 0.00 |
| *Latescibacteria* | 0.44 | 0.51 | 0.83 | 0.75 | 0.69 | 0.53 | 0.41 | 0.33 |
| *Microgenomates* | 0.00 | 0.00 | 0.00 | 0.00 | 0.01 | 0.03 | 0.01 | 0.02 |
| *Nitrospirae* | 1.00 | 1.27 | 1.78 | 1.50 | 1.93 | 1.82 | 1.02 | 0.91 |
| *Planctomycetes* | 2.99 | 3.57 | 4.72 | 4.79 | 5.01 | 4.94 | 4.55 | 4.03 |
| *Proteobacteria* | 45.00 | 43.41 | 32.41 | 34.63 | 30.08 | 30.22 | 33.19 | 34.77 |
| *candidate division SHA-109* | 0.02 | 0.01 | 0.01 | 0.01 | 0.00 | 0.01 | 0.01 | 0.00 |
| *candidate division SM2F11* | 0.00 | 0.00 | 0.00 | 0.01 | 0.00 | 0.00 | 0.00 | 0.00 |
| *Saccharibacteria* | 0.00 | 0.00 | 0.04 | 0.07 | 0.01 | 0.02 | 0.01 | 0.00 |
| *candidate division TM6* | 0.01 | 0.00 | 0.03 | 0.00 | 0.01 | 0.01 | 0.00 | 0.00 |
| *Thermotogae* | 0.00 | 0.01 | 0.00 | 0.00 | 0.00 | 0.00 | 0.00 | 0.00 |
| *Verrucomicrobia* | 0.48 | 1.53 | 0.61 | 0.56 | 0.73 | 0.52 | 0.69 | 0.66 |
| *candidate division WCHB1-60* | 0.01 | 0.03 | 0.01 | 0.01 | 0.01 | 0.03 | 0.05 | 0.04 |
|  |  |  |  |  |  |  |  |  |

Table S2 Relative abundances of bacterial class in the studied soils.

Note：Values represent percentages of all sequences assigned to the bacteria for soils.

| **Class** | **MM-1** | **MM-2** | **IM-1** | **IM-2** | **MA-1** | **MA-2** | **IA-1** | **IA-2** |
| --- | --- | --- | --- | --- | --- | --- | --- | --- |
| *Acidobacteria* | 14.92 | 13.81 | 22.48 | 20.91 | 21.20 | 22.22 | 18.40 | 17.82 |
| *Actinobacteria* | 16.25 | 15.68 | 13.70 | 14.19 | 17.12 | 16.62 | 17.47 | 17.70 |
| *Aerophobetes_norank* | 0.00 | 0.00 | 0.00 | 0.01 | 0.00 | 0.00 | 0.00 | 0.00 |
| *Alphaproteobacteria* | 13.04 | 15.07 | 9.79 | 9.78 | 10.12 | 9.42 | 10.77 | 11.35 |
| *Anaerolineae* | 1.77 | 1.86 | 1.98 | 1.74 | 1.85 | 1.71 | 1.42 | 1.51 |
| *Ardenticatenia* | 0.01 | 0.00 | 0.01 | 0.00 | 0.00 | 0.01 | 0.01 | 0.00 |
| *Armatimonadetes_norank* | 0.18 | 0.23 | 0.42 | 0.30 | 0.42 | 0.40 | 0.37 | 0.36 |
| *Armatimonadia* | 0.04 | 0.02 | 0.04 | 0.05 | 0.05 | 0.05 | 0.06 | 0.07 |
| *BD7-11* | 0.01 | 0.00 | 0.01 | 0.00 | 0.00 | 0.01 | 0.01 | 0.00 |
| *Bacilli* | 0.73 | 0.73 | 0.52 | 0.50 | 0.86 | 0.90 | 0.36 | 0.34 |
| *Bacteria_Unclassified* | 0.09 | 0.07 | 0.17 | 0.14 | 0.15 | 0.14 | 0.12 | 0.11 |
| *Bacteroidetes_Unclassified* | 0.00 | 0.00 | 0.00 | 0.00 | 0.01 | 0.00 | 0.00 | 0.00 |
| *Bacteroidia* | 0.03 | 0.00 | 0.00 | 0.00 | 0.00 | 0.00 | 0.00 | 0.00 |
| *Betaproteobacteria* | 8.76 | 5.22 | 11.21 | 10.41 | 11.28 | 12.04 | 10.72 | 10.40 |
| *C47* | 0.00 | 0.00 | 0.00 | 0.01 | 0.03 | 0.01 | 0.00 | 0.00 |
| *Caldilineae* | 0.03 | 0.06 | 0.08 | 0.05 | 0.07 | 0.09 | 0.05 | 0.07 |
| *Caldisericia* | 0.00 | 0.02 | 0.00 | 0.00 | 0.00 | 0.00 | 0.00 | 0.00 |
| *Chlamydiae* | 0.00 | 0.01 | 0.00 | 0.00 | 0.00 | 0.00 | 0.00 | 0.00 |
| *Chlorobia* | 0.07 | 0.07 | 0.09 | 0.10 | 0.13 | 0.07 | 0.14 | 0.18 |
| *Chloroflexi_Unclassified* | 0.00 | 0.01 | 0.00 | 0.00 | 0.01 | 0.01 | 0.00 | 0.00 |
| *Chloroflexi_norank* | 0.00 | 0.01 | 0.02 | 0.01 | 0.02 | 0.01 | 0.01 | 0.01 |
| *Chloroflexi_uncultured* | 0.03 | 0.01 | 0.01 | 0.02 | 0.04 | 0.04 | 0.01 | 0.00 |
| *Chloroflexia* | 1.08 | 1.13 | 1.31 | 1.20 | 1.09 | 1.20 | 1.19 | 1.18 |
| *Chthonomonadetes* | 0.21 | 0.25 | 0.28 | 0.26 | 0.34 | 0.33 | 0.25 | 0.29 |
| *Clostridia* | 0.03 | 0.04 | 0.02 | 0.02 | 0.01 | 0.00 | 0.02 | 0.03 |
| *Cyanobacteria* | 0.04 | 0.03 | 0.01 | 0.01 | 0.08 | 0.04 | 0.14 | 0.13 |
| *Cytophagia* | 0.74 | 0.70 | 0.72 | 0.54 | 0.45 | 0.48 | 1.02 | 1.05 |
| *Deinococci* | 0.00 | 0.00 | 0.00 | 0.00 | 0.00 | 0.00 | 0.00 | 0.01 |
| *Deltaproteobacteria* | 3.51 | 3.81 | 4.88 | 5.10 | 4.57 | 4.55 | 6.42 | 6.76 |
| *Elev-16S-509* | 0.02 | 0.01 | 0.01 | 0.01 | 0.01 | 0.01 | 0.00 | 0.00 |
| *Elusimicrobia* | 0.01 | 0.01 | 0.04 | 0.03 | 0.02 | 0.07 | 0.07 | 0.06 |
| *Erysipelotrichia* | 0.01 | 0.00 | 0.00 | 0.00 | 0.00 | 0.00 | 0.00 | 0.00 |
| *Flavobacteriia* | 0.06 | 0.06 | 0.17 | 0.12 | 0.21 | 0.15 | 0.21 | 0.08 |
| *Gammaproteobacteria* | 19.67 | 19.31 | 6.46 | 9.32 | 4.09 | 4.21 | 5.29 | 6.25 |
| *Gemmatimonadetes* | 6.58 | 8.05 | 9.96 | 9.92 | 9.64 | 10.03 | 11.49 | 11.18 |
| *Gitt-GS-136* | 0.38 | 0.31 | 0.43 | 0.39 | 0.46 | 0.54 | 0.17 | 0.25 |
| *Hydrogenedentes_norank* | 0.03 | 0.00 | 0.03 | 0.02 | 0.03 | 0.02 | 0.01 | 0.00 |
| *JG30-KF-CM66* | 0.04 | 0.07 | 0.06 | 0.08 | 0.09 | 0.08 | 0.04 | 0.05 |
| *KD4-96* | 0.75 | 0.90 | 1.06 | 1.05 | 0.92 | 0.79 | 0.70 | 0.70 |
| *Ktedonobacteria* | 0.55 | 0.35 | 0.54 | 0.50 | 0.41 | 0.46 | 0.33 | 0.29 |
| *Latescibacteria_norank* | 0.44 | 0.51 | 0.83 | 0.75 | 0.69 | 0.53 | 0.41 | 0.33 |
| *Microgenomates_norank* | 0.00 | 0.00 | 0.00 | 0.00 | 0.01 | 0.03 | 0.01 | 0.02 |
| *Negativicutes* | 0.02 | 0.00 | 0.00 | 0.00 | 0.00 | 0.00 | 0.01 | 0.00 |
| *Nitrospira* | 1.00 | 1.27 | 1.78 | 1.50 | 1.93 | 1.82 | 1.02 | 0.91 |
| *OM190* | 0.11 | 0.15 | 0.21 | 0.28 | 0.21 | 0.28 | 0.13 | 0.14 |
| *OPB35 soil group* | 0.06 | 0.21 | 0.07 | 0.03 | 0.05 | 0.04 | 0.03 | 0.07 |
| *Opitutae* | 0.20 | 0.30 | 0.27 | 0.27 | 0.36 | 0.27 | 0.56 | 0.46 |
| *P2-11E* | 0.29 | 0.42 | 0.31 | 0.34 | 0.46 | 0.41 | 0.10 | 0.09 |
| *Phycisphaerae* | 1.82 | 2.07 | 3.10 | 3.03 | 2.90 | 2.74 | 2.85 | 2.42 |
| *Pla3 lineage* | 0.00 | 0.00 | 0.00 | 0.00 | 0.01 | 0.00 | 0.00 | 0.00 |
| *Pla4 lineage* | 0.03 | 0.06 | 0.02 | 0.04 | 0.05 | 0.04 | 0.06 | 0.02 |
| *Planctomycetacia* | 1.02 | 1.27 | 1.35 | 1.42 | 1.80 | 1.85 | 1.46 | 1.42 |
| *Planctomycetes_Unclassified* | 0.01 | 0.01 | 0.00 | 0.00 | 0.01 | 0.00 | 0.00 | 0.00 |
| *Proteobacteria_Unclassified* | 0.00 | 0.00 | 0.05 | 0.01 | 0.01 | 0.00 | 0.00 | 0.00 |
| *S085* | 0.04 | 0.03 | 0.03 | 0.09 | 0.04 | 0.04 | 0.01 | 0.08 |
| *SHA-109_norank* | 0.02 | 0.01 | 0.01 | 0.01 | 0.00 | 0.01 | 0.01 | 0.00 |
| *SHA-26* | 0.00 | 0.01 | 0.01 | 0.01 | 0.01 | 0.02 | 0.01 | 0.01 |
| *SM2F11_norank* | 0.00 | 0.00 | 0.00 | 0.01 | 0.00 | 0.00 | 0.00 | 0.00 |
| *Saccharibacteria_norank* | 0.00 | 0.00 | 0.04 | 0.07 | 0.01 | 0.02 | 0.01 | 0.00 |
| *Spartobacteria* | 0.22 | 1.02 | 0.27 | 0.26 | 0.32 | 0.21 | 0.10 | 0.13 |
| *Sphingobacteriia* | 3.43 | 3.26 | 3.33 | 3.10 | 3.07 | 2.77 | 4.51 | 4.04 |
| *TK10* | 1.09 | 0.84 | 1.16 | 1.23 | 1.37 | 1.41 | 0.73 | 0.82 |
| *TM6_norank* | 0.01 | 0.00 | 0.03 | 0.00 | 0.01 | 0.01 | 0.00 | 0.00 |
| *Thermomicrobia* | 0.52 | 0.58 | 0.56 | 0.71 | 0.88 | 0.77 | 0.65 | 0.76 |
| *Thermotogae* | 0.00 | 0.01 | 0.00 | 0.00 | 0.00 | 0.00 | 0.00 | 0.00 |
| *Verrucomicrobia_Unclassified* | 0.00 | 0.01 | 0.00 | 0.00 | 0.00 | 0.00 | 0.00 | 0.00 |
| *WCHB1-60_norank* | 0.01 | 0.03 | 0.01 | 0.01 | 0.01 | 0.03 | 0.05 | 0.04 |
| *vadinHA49* | 0.00 | 0.01 | 0.01 | 0.00 | 0.01 | 0.01 | 0.05 | 0.03 |

Table S3 Relative abundances of bacterial genus in the studied soils.

Note：Values represent percentages of all sequences assigned to the bacteria for soils.

| **Genus** | **MM-1** | | | **MM-2** | **IM-1** | | **IM-2** | | **MA-1** | | **MA-2** | | **IA-1** | | **IA-2** |
| --- | --- | --- | --- | --- | --- | --- | --- | --- | --- | --- | --- | --- | --- | --- | --- |
| *0319-6M6_norank* | | 0.3168 | 0.1731 | | | 0.1952 | | 0.2947 | | 0.2542 | | 0.3094 | | 0.3610 | 0.3352 |
| *08D2Z23_norank* | | 0.0000 | 0.0221 | | | 0.0000 | | 0.0000 | | 0.0037 | | 0.0037 | | 0.0000 | 0.0000 |
| *11-24_norank* | | 0.3352 | 0.1768 | | | 0.4715 | | 0.4310 | | 0.4236 | | 0.5157 | | 0.3278 | 0.2873 |
| *288-2_norank* | | 0.4899 | 0.2984 | | | 0.4347 | | 0.4899 | | 0.3978 | | 0.4347 | | 0.5120 | 0.4678 |
| *480-2_norank* | | 0.3647 | 0.4457 | | | 0.3463 | | 0.3868 | | 0.4420 | | 0.4715 | | 0.3905 | 0.4236 |
| *A0839_norank* | | 0.0184 | 0.0184 | | | 0.0332 | | 0.0184 | | 0.0000 | | 0.0111 | | 0.0147 | 0.0221 |
| *ABS-19_norank* | | 0.2726 | 0.2173 | | | 0.4899 | | 0.4641 | | 0.4678 | | 0.3684 | | 0.8620 | 1.0241 |
| *AKIW1012_norank* | | 0.0147 | 0.0000 | | | 0.0000 | | 0.0000 | | 0.0000 | | 0.0000 | | 0.0000 | 0.0000 |
| *AKIW781_norank* | | 0.0442 | 0.0626 | | | 0.0847 | | 0.0847 | | 0.0774 | | 0.0921 | | 0.1289 | 0.1363 |
| *AKYG1722_norank* | | 0.0442 | 0.0442 | | | 0.0221 | | 0.0111 | | 0.0184 | | 0.0258 | | 0.0258 | 0.0295 |
| *AKYG587* | | 0.0221 | 0.0074 | | | 0.0074 | | 0.0000 | | 0.0147 | | 0.0147 | | 0.0037 | 0.0037 |
| *AKYH767_norank* | | 0.0516 | 0.0774 | | | 0.0995 | | 0.0847 | | 0.0774 | | 0.0663 | | 0.1326 | 0.1326 |
| *AT425-EubC11 terrestrial group_norank* | | 0.2615 | 0.2358 | | | 0.6373 | | 0.8472 | | 0.6410 | | 0.6704 | | 1.3151 | 1.3077 |
| *Acetobacteraceae_Unclassified* | | 0.0516 | 0.0553 | | | 0.0442 | | 0.0589 | | 0.0442 | | 0.0368 | | 0.0921 | 0.1031 |
| *Acetobacteraceae_uncultured* | | 0.0589 | 0.0221 | | | 0.0405 | | 0.0442 | | 0.0368 | | 0.0553 | | 0.1547 | 0.1547 |
| *Acidibacter* | | 0.9577 | 0.5968 | | | 0.9246 | | 0.8436 | | 0.5415 | | 0.5304 | | 1.3408 | 1.2709 |
| *Acidimicrobiaceae_uncultured* | | 0.0995 | 0.1179 | | | 0.1731 | | 0.1142 | | 0.1805 | | 0.1068 | | 0.0663 | 0.0884 |
| *Acidimicrobiales_Unclassified* | | 0.0111 | 0.0147 | | | 0.0332 | | 0.0332 | | 0.1105 | | 0.1694 | | 0.1326 | 0.1252 |
| *Acidimicrobiales_norank* | | 0.0037 | 0.0037 | | | 0.0221 | | 0.0184 | | 0.0295 | | 0.0111 | | 0.0111 | 0.0184 |
| *Acidimicrobiales_uncultured* | | 0.6004 | 0.5341 | | | 0.7994 | | 0.7699 | | 1.0204 | | 0.8767 | | 0.6225 | 0.8104 |
| *Acidobacteria_norank* | | 0.0847 | 0.0774 | | | 0.1031 | | 0.0958 | | 0.1142 | | 0.1694 | | 0.0700 | 0.0553 |
| *Acidobacteriaceae (Subgroup 1)_uncultured* | | 0.0479 | 0.1068 | | | 0.3831 | | 0.2836 | | 0.2358 | | 0.2615 | | 0.1216 | 0.1768 |
| *Acidobacterium* | | 0.1694 | 0.1547 | | | 0.0000 | | 0.0000 | | 0.0000 | | 0.0000 | | 0.0074 | 0.0000 |
| *Acidothermus* | | 0.0037 | 0.0221 | | | 0.0000 | | 0.0000 | | 0.0074 | | 0.0000 | | 0.0000 | 0.0037 |
| *Acinetobacter* | | 0.0405 | 0.0295 | | | 0.0074 | | 0.0368 | | 0.0074 | | 0.0074 | | 0.0000 | 0.0147 |
| *Actinobacteria_Unclassified* | | 0.0737 | 0.1068 | | | 0.0737 | | 0.1142 | | 0.0958 | | 0.0847 | | 0.0737 | 0.0995 |
| *Actinobacteria_norank* | | 0.8914 | 0.8030 | | | 0.9062 | | 0.9835 | | 1.0020 | | 1.0904 | | 0.6299 | 0.6631 |
| *Actinocorallia* | | 0.0368 | 0.1031 | | | 0.0221 | | 0.0479 | | 0.0295 | | 0.0147 | | 0.0405 | 0.0442 |
| *Actinomadura* | | 0.0442 | 0.0700 | | | 0.0037 | | 0.0074 | | 0.0000 | | 0.0147 | | 0.0111 | 0.0037 |
| *Actinophytocola* | | 0.0000 | 0.0184 | | | 0.0000 | | 0.0037 | | 0.0000 | | 0.0037 | | 0.0000 | 0.0000 |
| *Actinoplanes* | | 0.2100 | 0.1437 | | | 0.1768 | | 0.1768 | | 0.1915 | | 0.1473 | | 0.3057 | 0.2394 |
| *Adhaeribacter* | | 0.0368 | 0.0368 | | | 0.0663 | | 0.0589 | | 0.0479 | | 0.0700 | | 0.0958 | 0.0516 |
| *Aeromicrobium* | | 0.0884 | 0.1621 | | | 0.0700 | | 0.0810 | | 0.1473 | | 0.1731 | | 0.1510 | 0.1658 |
| *Aerophobetes_norank* | | 0.0000 | 0.0000 | | | 0.0037 | | 0.0074 | | 0.0037 | | 0.0000 | | 0.0000 | 0.0000 |
| *Afipia* | | 0.0332 | 0.0995 | | | 0.0221 | | 0.0184 | | 0.0111 | | 0.0184 | | 0.0479 | 0.0479 |
| *Agromyces* | | 0.0921 | 0.0479 | | | 0.1252 | | 0.1694 | | 0.1289 | | 0.1105 | | 0.1252 | 0.1473 |
| *Alcaligenaceae_Unclassified* | | 0.0626 | 0.0147 | | | 0.0000 | | 0.0000 | | 0.0000 | | 0.0000 | | 0.0000 | 0.0000 |
| *Alcaligenaceae_uncultured* | | 0.0626 | 0.0000 | | | 0.0553 | | 0.0810 | | 0.0626 | | 0.0626 | | 0.0295 | 0.0332 |
| *Aliihoeflea* | | 0.3499 | 0.3647 | | | 0.0663 | | 0.1952 | | 0.0479 | | 0.0921 | | 0.0221 | 0.0626 |
| *Alphaproteobacteria_Unclassified* | | 0.0147 | 0.0405 | | | 0.0332 | | 0.0442 | | 0.0221 | | 0.0295 | | 0.0663 | 0.0626 |
| *Altererythrobacter* | | 0.1658 | 0.3057 | | | 0.2984 | | 0.3094 | | 0.3094 | | 0.2579 | | 0.6631 | 0.6483 |
| *Aminobacter* | | 0.0258 | 0.0295 | | | 0.0074 | | 0.0000 | | 0.0074 | | 0.0074 | | 0.0184 | 0.0111 |
| *Amycolatopsis* | | 0.0626 | 0.0368 | | | 0.0147 | | 0.0147 | | 0.0368 | | 0.0479 | | 0.0516 | 0.0553 |
| *Anaerolinea* | | 0.0000 | 0.0000 | | | 0.0000 | | 0.0111 | | 0.0000 | | 0.0000 | | 0.0000 | 0.0000 |
| *Anaerolineaceae_uncultured* | | 1.7682 | 1.8602 | | | 1.9818 | | 1.7276 | | 1.8455 | | 1.7055 | | 1.4182 | 1.5066 |
| *Anaeromyxobacter* | | 0.0295 | 0.0147 | | | 0.0332 | | 0.0332 | | 0.0295 | | 0.0295 | | 0.0442 | 0.0111 |
| *Angustibacter* | | 0.3573 | 0.4310 | | | 0.0111 | | 0.0000 | | 0.0037 | | 0.0111 | | 0.0221 | 0.0037 |
| *Aquicella* | | 0.1989 | 0.3647 | | | 0.1326 | | 0.1621 | | 0.0663 | | 0.0958 | | 0.0405 | 0.0405 |
| *Ardenticatenia_uncultured* | | 0.0074 | 0.0000 | | | 0.0111 | | 0.0037 | | 0.0000 | | 0.0074 | | 0.0074 | 0.0037 |
| *Arenimonas* | | 0.8583 | 0.8767 | | | 0.4678 | | 0.4420 | | 0.4199 | | 0.3684 | | 0.8730 | 0.8804 |
| *Armatimonadales_norank* | | 0.0332 | 0.0221 | | | 0.0111 | | 0.0332 | | 0.0405 | | 0.0368 | | 0.0516 | 0.0589 |
| *Armatimonadetes_norank* | | 0.1805 | 0.2284 | | | 0.4163 | | 0.3021 | | 0.4236 | | 0.3978 | | 0.3720 | 0.3573 |
| *Armatimonas* | | 0.0037 | 0.0000 | | | 0.0332 | | 0.0147 | | 0.0074 | | 0.0111 | | 0.0111 | 0.0074 |
| *Arthrobacter* | | 0.7625 | 0.7146 | | | 0.7515 | | 0.6778 | | 1.5692 | | 1.3629 | | 0.9835 | 0.9541 |
| *Asticcacaulis* | | 0.0479 | 0.0884 | | | 0.0037 | | 0.0000 | | 0.0000 | | 0.0000 | | 0.0037 | 0.0184 |
| *Azospirillum* | | 0.0000 | 0.0000 | | | 0.0074 | | 0.0000 | | 0.0037 | | 0.0037 | | 0.0000 | 0.0037 |
| *B1-7BS_norank* | | 0.0000 | 0.0074 | | | 0.0000 | | 0.0000 | | 0.0000 | | 0.0111 | | 0.0111 | 0.0111 |
| *BCf3-20_norank* | | 0.0700 | 0.0958 | | | 0.0516 | | 0.0368 | | 0.0147 | | 0.0368 | | 0.0553 | 0.0405 |
| *BD2-11 terrestrial group_norank* | | 0.0184 | 0.0184 | | | 0.0258 | | 0.0295 | | 0.0626 | | 0.0368 | | 0.0368 | 0.0184 |
| *BD7-11_norank* | | 0.0074 | 0.0037 | | | 0.0074 | | 0.0037 | | 0.0000 | | 0.0111 | | 0.0111 | 0.0037 |
| *BIrii41_norank* | | 0.0405 | 0.0332 | | | 0.1879 | | 0.1768 | | 0.1068 | | 0.1473 | | 1.3666 | 1.2709 |
| *Bacillaceae_Unclassified* | | 0.0000 | 0.0111 | | | 0.0000 | | 0.0000 | | 0.0000 | | 0.0000 | | 0.0000 | 0.0000 |
| *Bacillus* | | 0.2505 | 0.3352 | | | 0.3315 | | 0.3021 | | 0.4384 | | 0.5341 | | 0.2137 | 0.1658 |
| *Bacteria_Unclassified* | | 0.0921 | 0.0700 | | | 0.1731 | | 0.1400 | | 0.1510 | | 0.1437 | | 0.1179 | 0.1068 |
| *Bacteroidetes_Unclassified* | | 0.0000 | 0.0037 | | | 0.0000 | | 0.0000 | | 0.0111 | | 0.0037 | | 0.0000 | 0.0000 |
| *Bauldia* | | 0.0221 | 0.0516 | | | 0.0958 | | 0.0479 | | 0.0368 | | 0.0589 | | 0.0368 | 0.0516 |
| *Betaproteobacteria_Unclassified* | | 0.0147 | 0.0147 | | | 0.0479 | | 0.0921 | | 0.0295 | | 0.0626 | | 0.0516 | 0.0295 |
| *Blastocatella* | | 0.7736 | 0.4605 | | | 1.1972 | | 1.0130 | | 0.9062 | | 0.9062 | | 1.1898 | 1.0425 |
| *Blastococcus* | | 0.9172 | 0.8251 | | | 0.9099 | | 1.0314 | | 1.0462 | | 0.9356 | | 1.7755 | 1.8124 |
| *Blfdi19_norank* | | 0.0000 | 0.0000 | | | 0.0111 | | 0.0111 | | 0.0184 | | 0.0074 | | 0.0184 | 0.0147 |
| *Bordetella* | | 0.0184 | 0.0111 | | | 0.0000 | | 0.0037 | | 0.0037 | | 0.0037 | | 0.0147 | 0.0111 |
| *Bosea* | | 0.0147 | 0.0037 | | | 0.0037 | | 0.0184 | | 0.0221 | | 0.0184 | | 0.0589 | 0.0626 |
| *Bradyrhizobium* | | 1.2119 | 1.1088 | | | 0.6815 | | 0.7183 | | 0.8251 | | 0.7994 | | 0.9725 | 0.9946 |
| *Brevibacillus* | | 0.0000 | 0.0000 | | | 0.0000 | | 0.0111 | | 0.0000 | | 0.0037 | | 0.0000 | 0.0000 |
| *Brevundimonas* | | 0.0074 | 0.0037 | | | 0.0000 | | 0.0037 | | 0.0184 | | 0.0111 | | 0.0111 | 0.0221 |
| *Bryobacter* | | 0.6520 | 0.7257 | | | 0.8620 | | 0.6594 | | 1.0204 | | 1.0646 | | 0.9246 | 0.9356 |
| *Burkholderia* | | 0.0810 | 0.0111 | | | 0.0295 | | 0.0295 | | 0.0258 | | 0.0037 | | 0.0332 | 0.0479 |
| *Byssovorax* | | 0.0737 | 0.0589 | | | 0.1326 | | 0.0663 | | 0.1142 | | 0.1031 | | 0.1584 | 0.1584 |
| *C0119_norank* | | 0.5304 | 0.3389 | | | 0.5341 | | 0.4973 | | 0.4052 | | 0.4494 | | 0.3278 | 0.2763 |
| *C47_norank* | | 0.0000 | 0.0000 | | | 0.0000 | | 0.0074 | | 0.0258 | | 0.0111 | | 0.0000 | 0.0000 |
| *CA002_norank* | | 0.0000 | 0.0000 | | | 0.0000 | | 0.0000 | | 0.0147 | | 0.0037 | | 0.0258 | 0.0074 |
| *CL500-29 marine group* | | 0.1142 | 0.1105 | | | 0.2137 | | 0.1179 | | 0.1842 | | 0.1363 | | 0.1326 | 0.1731 |
| *CL500-3* | | 0.0000 | 0.0000 | | | 0.0037 | | 0.0000 | | 0.0000 | | 0.0037 | | 0.0074 | 0.0000 |
| *CPla-3 termite group_norank* | | 0.0405 | 0.0626 | | | 0.0368 | | 0.0589 | | 0.0479 | | 0.0958 | | 0.0847 | 0.0516 |
| *Caldilineaceae_Unclassified* | | 0.0037 | 0.0295 | | | 0.0147 | | 0.0147 | | 0.0332 | | 0.0332 | | 0.0147 | 0.0221 |
| *Caldilineaceae_uncultured* | | 0.0000 | 0.0000 | | | 0.0332 | | 0.0258 | | 0.0147 | | 0.0295 | | 0.0295 | 0.0368 |
| *Camelimonas* | | 0.0000 | 0.0000 | | | 0.0000 | | 0.0000 | | 0.0000 | | 0.0000 | | 0.0111 | 0.0000 |
| *Candidatus Alysiosphaera* | | 0.0368 | 0.0295 | | | 0.0516 | | 0.0663 | | 0.0663 | | 0.0663 | | 0.1105 | 0.1105 |
| *Candidatus Entotheonella* | | 0.2984 | 0.3573 | | | 0.4384 | | 0.5673 | | 0.4384 | | 0.4973 | | 0.3463 | 0.3463 |
| *Candidatus Koribacter* | | 0.0111 | 0.0221 | | | 0.0368 | | 0.0184 | | 0.0074 | | 0.0553 | | 0.0147 | 0.0111 |
| *Candidatus Microthrix* | | 0.0037 | 0.0111 | | | 0.0000 | | 0.0000 | | 0.0037 | | 0.0074 | | 0.0221 | 0.0111 |
| *Candidatus Solibacter* | | 0.3720 | 0.4273 | | | 0.3831 | | 0.3684 | | 0.4678 | | 0.5525 | | 0.4494 | 0.3536 |
| *Candidatus Xiphinematobacter* | | 0.0037 | 0.0037 | | | 0.0000 | | 0.0000 | | 0.0037 | | 0.0000 | | 0.0000 | 0.0000 |
| *Candidimonas* | | 0.1842 | 0.1142 | | | 0.0000 | | 0.0037 | | 0.0000 | | 0.0037 | | 0.0000 | 0.0000 |
| *Castellaniella* | | 0.0884 | 0.0737 | | | 0.0000 | | 0.0000 | | 0.0000 | | 0.0000 | | 0.0037 | 0.0074 |
| *Caulobacter* | | 0.1805 | 0.0774 | | | 0.0884 | | 0.0774 | | 0.0663 | | 0.0663 | | 0.1547 | 0.1842 |
| *Caulobacteraceae_Unclassified* | | 0.0847 | 0.1289 | | | 0.0589 | | 0.1068 | | 0.0589 | | 0.0479 | | 0.1252 | 0.0663 |
| *Caulobacteraceae_uncultured* | | 0.0442 | 0.0810 | | | 0.0405 | | 0.0516 | | 0.0737 | | 0.0774 | | 0.2100 | 0.1768 |
| *Cellulomonas* | | 0.0037 | 0.0074 | | | 0.0000 | | 0.0037 | | 0.0037 | | 0.0000 | | 0.0037 | 0.0074 |
| *Cellvibrio* | | 0.0000 | 0.0000 | | | 0.0000 | | 0.0037 | | 0.0000 | | 0.0000 | | 0.0332 | 0.0295 |
| *Chitinophaga* | | 0.0921 | 0.0737 | | | 0.1105 | | 0.0700 | | 0.1031 | | 0.1142 | | 0.2210 | 0.2063 |
| *Chitinophagaceae_Unclassified* | | 0.6078 | 0.5489 | | | 0.5010 | | 0.4862 | | 0.3168 | | 0.3463 | | 0.5304 | 0.3794 |
| *Chitinophagaceae_uncultured* | | 1.4919 | 1.4366 | | | 0.7257 | | 0.6520 | | 0.7588 | | 0.7257 | | 0.8030 | 0.5931 |
| *Chloroflexi_Unclassified* | | 0.0000 | 0.0111 | | | 0.0000 | | 0.0000 | | 0.0147 | | 0.0074 | | 0.0037 | 0.0000 |
| *Chloroflexi_norank* | | 0.0000 | 0.0147 | | | 0.0221 | | 0.0147 | | 0.0184 | | 0.0111 | | 0.0074 | 0.0111 |
| *Chloroflexi_uncultured* | | 0.0258 | 0.0147 | | | 0.0147 | | 0.0221 | | 0.0442 | | 0.0405 | | 0.0147 | 0.0037 |
| *Chryseolinea* | | 0.4420 | 0.3720 | | | 0.3426 | | 0.2763 | | 0.1363 | | 0.1400 | | 0.3131 | 0.3757 |
| *Chthoniobacter* | | 0.0332 | 0.0995 | | | 0.0147 | | 0.0147 | | 0.0442 | | 0.0111 | | 0.0295 | 0.0332 |
| *Chthonomonadales_norank* | | 0.0442 | 0.0884 | | | 0.0589 | | 0.0626 | | 0.1473 | | 0.1068 | | 0.0884 | 0.1473 |
| *Chthonomonas* | | 0.1621 | 0.1584 | | | 0.2210 | | 0.1989 | | 0.1879 | | 0.2210 | | 0.1621 | 0.1437 |
| *Clostridium sensu stricto 1* | | 0.0000 | 0.0111 | | | 0.0000 | | 0.0074 | | 0.0000 | | 0.0000 | | 0.0111 | 0.0074 |
| *Clostridium sensu stricto 12* | | 0.0037 | 0.0037 | | | 0.0037 | | 0.0000 | | 0.0037 | | 0.0037 | | 0.0074 | 0.0147 |
| *Cohnella* | | 0.0589 | 0.0442 | | | 0.0074 | | 0.0184 | | 0.0479 | | 0.0221 | | 0.0074 | 0.0184 |
| *Comamonadaceae_Unclassified* | | 0.6778 | 0.4457 | | | 0.6262 | | 0.5378 | | 0.8583 | | 0.8583 | | 0.9504 | 1.0609 |
| *Constrictibacter* | | 0.0111 | 0.0037 | | | 0.0037 | | 0.0000 | | 0.0037 | | 0.0037 | | 0.0000 | 0.0111 |
| *Coxiellaceae_uncultured* | | 0.0000 | 0.0000 | | | 0.0000 | | 0.0000 | | 0.0000 | | 0.0074 | | 0.0037 | 0.0000 |
| *Craurococcus* | | 0.0037 | 0.0037 | | | 0.0037 | | 0.0332 | | 0.0184 | | 0.0111 | | 0.0111 | 0.0147 |
| *Cryptosporangium* | | 0.0000 | 0.0000 | | | 0.0074 | | 0.0037 | | 0.0184 | | 0.0111 | | 0.0147 | 0.0147 |
| *Cupriavidus* | | 0.0000 | 0.0000 | | | 0.0184 | | 0.0000 | | 0.0184 | | 0.0037 | | 0.0074 | 0.0147 |
| *Cyanobacteria_Unclassified* | | 0.0000 | 0.0000 | | | 0.0037 | | 0.0074 | | 0.0000 | | 0.0074 | | 0.0074 | 0.0037 |
| *Cyanobacteria_norank* | | 0.0405 | 0.0111 | | | 0.0037 | | 0.0037 | | 0.0295 | | 0.0037 | | 0.0774 | 0.0589 |
| *Cystobacteraceae_Unclassified* | | 0.2284 | 0.2505 | | | 0.3021 | | 0.3536 | | 0.3905 | | 0.3905 | | 1.3851 | 1.5987 |
| *Cytophagaceae_Unclassified* | | 0.0405 | 0.0368 | | | 0.0553 | | 0.0442 | | 0.0479 | | 0.0405 | | 0.1179 | 0.1584 |
| *Cytophagaceae_uncultured* | | 0.1437 | 0.1731 | | | 0.1031 | | 0.0553 | | 0.0626 | | 0.0995 | | 0.1289 | 0.1031 |
| *DA101 soil group_norank* | | 0.1879 | 0.9172 | | | 0.2579 | | 0.2468 | | 0.2763 | | 0.2026 | | 0.0700 | 0.0995 |
| *DA111_norank* | | 0.3573 | 0.3905 | | | 0.4568 | | 0.4826 | | 0.4973 | | 0.3978 | | 0.2726 | 0.2873 |
| *DB1-14_norank* | | 0.0074 | 0.0074 | | | 0.0000 | | 0.0000 | | 0.0000 | | 0.0000 | | 0.0000 | 0.0000 |
| *DS-100_norank* | | 0.0995 | 0.0700 | | | 0.2247 | | 0.2247 | | 0.1658 | | 0.1879 | | 0.1731 | 0.1658 |
| *DUNssu044_norank* | | 0.0074 | 0.0184 | | | 0.0368 | | 0.0368 | | 0.0368 | | 0.0332 | | 0.0147 | 0.0332 |
| *Dactylosporangium* | | 0.0810 | 0.0553 | | | 0.0774 | | 0.0847 | | 0.0884 | | 0.0884 | | 0.1179 | 0.1252 |
| *Defluviicoccus* | | 0.0258 | 0.0074 | | | 0.0111 | | 0.0147 | | 0.0184 | | 0.0221 | | 0.0405 | 0.0258 |
| *Deinococcaceae_uncultured* | | 0.0000 | 0.0000 | | | 0.0000 | | 0.0000 | | 0.0000 | | 0.0000 | | 0.0037 | 0.0074 |
| *Deltaproteobacteria_Unclassified* | | 0.0000 | 0.0000 | | | 0.0000 | | 0.0000 | | 0.0000 | | 0.0000 | | 0.0000 | 0.0037 |
| *Desulfurellaceae_uncultured* | | 0.1437 | 0.1400 | | | 0.1510 | | 0.1437 | | 0.1473 | | 0.1879 | | 0.1915 | 0.1989 |
| *Devosia* | | 0.2947 | 0.3021 | | | 0.0516 | | 0.0516 | | 0.0774 | | 0.0737 | | 0.1473 | 0.1731 |
| *Dokdonella* | | 0.2726 | 0.2984 | | | 0.1142 | | 0.0810 | | 0.0553 | | 0.0516 | | 0.0884 | 0.0626 |
| *Dongia* | | 0.3352 | 0.4899 | | | 0.0995 | | 0.1289 | | 0.0700 | | 0.0663 | | 0.1915 | 0.1731 |
| *EV818SWSAP88_norank* | | 0.0147 | 0.0000 | | | 0.0000 | | 0.0000 | | 0.0000 | | 0.0000 | | 0.0000 | 0.0000 |
| *Edaphobacter* | | 0.0516 | 0.1473 | | | 0.0037 | | 0.0037 | | 0.0074 | | 0.0000 | | 0.0147 | 0.0000 |
| *Elev-16S-1166_norank* | | 0.0295 | 0.0147 | | | 0.0258 | | 0.0332 | | 0.0479 | | 0.0553 | | 0.0258 | 0.0111 |
| *Elev-16S-1332_norank* | | 0.0774 | 0.1068 | | | 0.0884 | | 0.0884 | | 0.1658 | | 0.1473 | | 0.1031 | 0.1473 |
| *Elev-16S-509_norank* | | 0.0184 | 0.0111 | | | 0.0147 | | 0.0074 | | 0.0111 | | 0.0111 | | 0.0037 | 0.0037 |
| *Ellin6055_norank* | | 0.0368 | 0.0516 | | | 0.0479 | | 0.0368 | | 0.0295 | | 0.0147 | | 0.0663 | 0.0737 |
| *Elusimicrobia_Unclassified* | | 0.0000 | 0.0074 | | | 0.0000 | | 0.0000 | | 0.0000 | | 0.0037 | | 0.0000 | 0.0000 |
| *Erysipelotrichaceae UCG-003* | | 0.0074 | 0.0000 | | | 0.0000 | | 0.0000 | | 0.0000 | | 0.0000 | | 0.0000 | 0.0000 |
| *FFCH13075_norank* | | 0.0405 | 0.0516 | | | 0.0810 | | 0.0847 | | 0.0774 | | 0.0774 | | 0.1363 | 0.0626 |
| *FFCH16767_norank* | | 0.0000 | 0.0000 | | | 0.0000 | | 0.0000 | | 0.0000 | | 0.0037 | | 0.0037 | 0.0111 |
| *FFCH5858_norank* | | 0.0111 | 0.0074 | | | 0.0111 | | 0.0074 | | 0.0111 | | 0.0074 | | 0.0074 | 0.0111 |
| *FFCH7168_norank* | | 0.0405 | 0.0332 | | | 0.0184 | | 0.0442 | | 0.0221 | | 0.0074 | | 0.0295 | 0.0295 |
| *Ferruginibacter* | | 0.4347 | 0.2468 | | | 0.8325 | | 0.7772 | | 0.7515 | | 0.6741 | | 1.2488 | 1.1567 |
| *Flavisolibacter* | | 0.1805 | 0.2321 | | | 0.4384 | | 0.3868 | | 0.4236 | | 0.3131 | | 0.5378 | 0.5525 |
| *Flavitalea* | | 0.1400 | 0.1621 | | | 0.2173 | | 0.2505 | | 0.1437 | | 0.1473 | | 0.2321 | 0.2542 |
| *Flavobacterium* | | 0.0516 | 0.0405 | | | 0.1658 | | 0.1179 | | 0.2100 | | 0.1400 | | 0.1768 | 0.0700 |
| *Frankia* | | 0.0626 | 0.1289 | | | 0.0553 | | 0.0589 | | 0.0663 | | 0.0884 | | 0.0332 | 0.0442 |
| *Frankiales_Unclassified* | | 0.0074 | 0.0037 | | | 0.0221 | | 0.0037 | | 0.0074 | | 0.0221 | | 0.0074 | 0.0037 |
| *Frankiales_uncultured* | | 0.0737 | 0.0700 | | | 0.0921 | | 0.0810 | | 0.3426 | | 0.2689 | | 0.3868 | 0.4310 |
| *GAL15* | | 0.0000 | 0.0074 | | | 0.0000 | | 0.0000 | | 0.0037 | | 0.0037 | | 0.0000 | 0.0000 |
| *GR-WP33-30_norank* | | 1.1530 | 1.3519 | | | 1.7903 | | 1.9523 | | 1.5434 | | 1.6540 | | 0.9283 | 1.1235 |
| *Gaiella* | | 1.4072 | 1.4514 | | | 1.5545 | | 1.5545 | | 1.6245 | | 1.6134 | | 1.2119 | 1.0646 |
| *Gaiellales_uncultured* | | 2.7406 | 2.5601 | | | 2.2323 | | 2.2581 | | 2.4680 | | 2.6449 | | 1.7276 | 1.6761 |
| *Galbitalea* | | 0.5673 | 0.6152 | | | 0.0405 | | 0.0405 | | 0.0442 | | 0.0553 | | 0.1326 | 0.1473 |
| *Geminicoccus* | | 0.0037 | 0.0147 | | | 0.0000 | | 0.0000 | | 0.0000 | | 0.0000 | | 0.0074 | 0.0147 |
| *Gemmata* | | 0.1400 | 0.2210 | | | 0.3021 | | 0.2579 | | 0.3242 | | 0.3242 | | 0.3094 | 0.2284 |
| *Gemmatimonadaceae_Unclassified* | | 0.2836 | 0.4568 | | | 0.5489 | | 0.4605 | | 0.5746 | | 0.6410 | | 0.8104 | 0.6962 |
| *Gemmatimonadaceae_uncultured* | | 3.3263 | 4.0594 | | | 4.8661 | | 4.9729 | | 4.4093 | | 4.3467 | | 3.8420 | 3.8973 |
| *Gemmatimonas* | | 2.6228 | 3.1974 | | | 3.7868 | | 3.5179 | | 3.8126 | | 4.2067 | | 5.3118 | 5.0761 |
| *Geodermatophilus* | | 0.0626 | 0.0516 | | | 0.0626 | | 0.0810 | | 0.0995 | | 0.0589 | | 0.1473 | 0.1031 |
| *Gitt-GS-136_norank* | | 0.3757 | 0.3094 | | | 0.4273 | | 0.3868 | | 0.4568 | | 0.5378 | | 0.1658 | 0.2505 |
| *Gracilibacter* | | 0.0000 | 0.0000 | | | 0.0111 | | 0.0000 | | 0.0000 | | 0.0000 | | 0.0000 | 0.0000 |
| *Granulicella* | | 0.2726 | 0.2652 | | | 0.0111 | | 0.0074 | | 0.0037 | | 0.0000 | | 0.0074 | 0.0000 |
| *Haliangium* | | 0.9062 | 1.0241 | | | 0.7515 | | 0.7183 | | 0.7846 | | 0.6704 | | 0.6262 | 0.5268 |
| *Haliea* | | 0.0111 | 0.0074 | | | 0.0258 | | 0.0184 | | 0.0000 | | 0.0074 | | 0.0111 | 0.0074 |
| *Halomonas* | | 5.5255 | 4.6377 | | | 0.5268 | | 3.2895 | | 0.5746 | | 0.8104 | | 0.1805 | 1.2598 |
| *Hamadaea* | | 0.0663 | 0.0626 | | | 0.0553 | | 0.0332 | | 0.0332 | | 0.0184 | | 0.0184 | 0.0405 |
| *Heliobacteriaceae_uncultured* | | 0.0258 | 0.0184 | | | 0.0000 | | 0.0000 | | 0.0037 | | 0.0000 | | 0.0000 | 0.0000 |
| *Herpetosiphon* | | 0.0516 | 0.0147 | | | 0.0000 | | 0.0037 | | 0.0037 | | 0.0074 | | 0.0295 | 0.0295 |
| *Hydrogenedentes_norank* | | 0.0258 | 0.0037 | | | 0.0295 | | 0.0221 | | 0.0258 | | 0.0184 | | 0.0111 | 0.0000 |
| *Hymenobacter* | | 0.0184 | 0.0111 | | | 0.0332 | | 0.0332 | | 0.0442 | | 0.0368 | | 0.0626 | 0.1105 |
| *Hyphomicrobium* | | 0.0111 | 0.0332 | | | 0.0221 | | 0.0184 | | 0.0111 | | 0.0037 | | 0.0074 | 0.0221 |
| *I-10_norank* | | 0.4310 | 0.4678 | | | 0.0958 | | 0.0737 | | 0.1142 | | 0.0810 | | 0.0737 | 0.0332 |
| *I-8* | | 0.0000 | 0.0000 | | | 0.0000 | | 0.0000 | | 0.0037 | | 0.0000 | | 0.0000 | 0.0000 |
| *Iamia* | | 0.0884 | 0.0847 | | | 0.1142 | | 0.1216 | | 0.1179 | | 0.0847 | | 0.1289 | 0.1510 |
| *Illumatobacter* | | 0.1216 | 0.1363 | | | 0.1216 | | 0.1915 | | 0.1252 | | 0.0884 | | 0.1289 | 0.1584 |
| *Inquilinus* | | 0.0737 | 0.0700 | | | 0.0037 | | 0.0184 | | 0.0295 | | 0.0000 | | 0.0111 | 0.0037 |
| *Intrasporangiaceae_Unclassified* | | 0.2800 | 0.2210 | | | 0.1952 | | 0.1547 | | 0.2063 | | 0.1842 | | 0.2358 | 0.1473 |
| *Isosphaera* | | 0.0000 | 0.0074 | | | 0.0037 | | 0.0000 | | 0.0037 | | 0.0074 | | 0.0000 | 0.0037 |
| *JG30-KF-AS9_norank* | | 0.0000 | 0.0000 | | | 0.0037 | | 0.0037 | | 0.0000 | | 0.0037 | | 0.0000 | 0.0000 |
| *JG30-KF-CM45_norank* | | 0.4605 | 0.5157 | | | 0.5341 | | 0.6925 | | 0.8620 | | 0.7404 | | 0.6262 | 0.7073 |
| *JG30-KF-CM66_norank* | | 0.0442 | 0.0663 | | | 0.0626 | | 0.0774 | | 0.0921 | | 0.0810 | | 0.0442 | 0.0479 |
| *JG34-KF-361_norank* | | 0.0589 | 0.0774 | | | 0.0663 | | 0.0774 | | 0.0479 | | 0.0737 | | 0.0295 | 0.0295 |
| *JG37-AG-20_norank* | | 0.0037 | 0.0037 | | | 0.0074 | | 0.0000 | | 0.0074 | | 0.0037 | | 0.0000 | 0.0074 |
| *Jatrophihabitans* | | 0.0516 | 0.0737 | | | 0.0553 | | 0.0553 | | 0.0774 | | 0.0884 | | 0.0589 | 0.1031 |
| *KCM-B-15_norank* | | 0.0405 | 0.0111 | | | 0.0221 | | 0.0221 | | 0.0074 | | 0.0037 | | 0.0258 | 0.0037 |
| *KCM-B-60_norank* | | 0.0074 | 0.0037 | | | 0.0221 | | 0.0184 | | 0.0074 | | 0.0147 | | 0.0000 | 0.0037 |
| *KD3-93_norank* | | 0.0368 | 0.0111 | | | 0.0147 | | 0.0111 | | 0.0074 | | 0.0074 | | 0.0074 | 0.0147 |
| *KD4-96_norank* | | 0.7478 | 0.9025 | | | 1.0646 | | 1.0462 | | 0.9172 | | 0.7883 | | 0.6962 | 0.6999 |
| *KF-JG30-B3_norank* | | 0.1879 | 0.1252 | | | 0.1289 | | 0.1621 | | 0.1584 | | 0.1437 | | 0.0958 | 0.0774 |
| *KI89A clade_norank* | | 0.0184 | 0.0258 | | | 0.0074 | | 0.0405 | | 0.0074 | | 0.0111 | | 0.0258 | 0.0332 |
| *Kineosporia* | | 0.0442 | 0.0626 | | | 0.0074 | | 0.0553 | | 0.0516 | | 0.0553 | | 0.1289 | 0.0958 |
| *Kribbella* | | 0.1989 | 0.2836 | | | 0.1731 | | 0.1252 | | 0.1768 | | 0.1363 | | 0.1584 | 0.1768 |
| *Ktedonobacteraceae_uncultured* | | 0.0074 | 0.0037 | | | 0.0000 | | 0.0000 | | 0.0000 | | 0.0000 | | 0.0000 | 0.0037 |
| *Labrys* | | 0.0111 | 0.0295 | | | 0.0368 | | 0.0147 | | 0.0295 | | 0.0479 | | 0.0368 | 0.0368 |
| *Lacibacter* | | 0.0000 | 0.0000 | | | 0.0000 | | 0.0037 | | 0.0037 | | 0.0000 | | 0.0147 | 0.0000 |
| *Lactococcus* | | 0.0147 | 0.0000 | | | 0.0000 | | 0.0000 | | 0.0000 | | 0.0037 | | 0.0000 | 0.0000 |
| *Latescibacteria_norank* | | 0.4420 | 0.5083 | | | 0.8288 | | 0.7515 | | 0.6888 | | 0.5268 | | 0.4089 | 0.3315 |
| *Lautropia* | | 0.0000 | 0.0000 | | | 0.0037 | | 0.0184 | | 0.0037 | | 0.0111 | | 0.0111 | 0.0037 |
| *Legionellales_Unclassified* | | 0.0000 | 0.0184 | | | 0.0000 | | 0.0000 | | 0.0000 | | 0.0000 | | 0.0000 | 0.0000 |
| *Leptolyngbya* | | 0.0000 | 0.0074 | | | 0.0000 | | 0.0000 | | 0.0037 | | 0.0000 | | 0.0074 | 0.0037 |
| *Lineage IIa_norank* | | 0.0000 | 0.0037 | | | 0.0147 | | 0.0074 | | 0.0147 | | 0.0221 | | 0.0295 | 0.0368 |
| *Lineage IIb_norank* | | 0.0000 | 0.0000 | | | 0.0074 | | 0.0074 | | 0.0037 | | 0.0332 | | 0.0442 | 0.0147 |
| *Lineage IIc_norank* | | 0.0074 | 0.0000 | | | 0.0037 | | 0.0000 | | 0.0000 | | 0.0037 | | 0.0000 | 0.0000 |
| *Lineage IV_norank* | | 0.0000 | 0.0000 | | | 0.0184 | | 0.0184 | | 0.0037 | | 0.0111 | | 0.0000 | 0.0074 |
| *Litorilinea* | | 0.0258 | 0.0332 | | | 0.0295 | | 0.0074 | | 0.0221 | | 0.0258 | | 0.0037 | 0.0074 |
| *Luedemannella* | | 0.1731 | 0.2247 | | | 0.1216 | | 0.1731 | | 0.1142 | | 0.0663 | | 0.0663 | 0.0774 |
| *Luteibacter* | | 0.1437 | 0.2100 | | | 0.0000 | | 0.0147 | | 0.0000 | | 0.0000 | | 0.0147 | 0.0184 |
| *Lysobacter* | | 0.9356 | 1.0756 | | | 1.4698 | | 1.5434 | | 0.6115 | | 0.5047 | | 0.7109 | 0.6336 |
| *ML80_norank* | | 0.0000 | 0.0258 | | | 0.0074 | | 0.0074 | | 0.0111 | | 0.0111 | | 0.0074 | 0.0074 |
| *MNC12_norank* | | 0.0074 | 0.0147 | | | 0.0884 | | 0.0553 | | 0.0442 | | 0.0332 | | 0.0221 | 0.0295 |
| *MND8_norank* | | 0.0111 | 0.0184 | | | 0.0111 | | 0.0111 | | 0.0221 | | 0.0221 | | 0.0111 | 0.0000 |
| *MNG7_norank* | | 0.0147 | 0.0000 | | | 0.0258 | | 0.0295 | | 0.0184 | | 0.0111 | | 0.0405 | 0.0442 |
| *MSB-1E8_norank* | | 0.0553 | 0.0405 | | | 0.0553 | | 0.0368 | | 0.0516 | | 0.0479 | | 0.0589 | 0.0221 |
| *Marmoricola* | | 0.4605 | 0.4052 | | | 0.2137 | | 0.2615 | | 0.4236 | | 0.3868 | | 0.4936 | 0.5673 |
| *Massilia* | | 0.2210 | 0.1179 | | | 0.3352 | | 0.2468 | | 0.4605 | | 0.4347 | | 0.6262 | 0.5636 |
| *Meganema* | | 0.0111 | 0.0295 | | | 0.0000 | | 0.0000 | | 0.0000 | | 0.0000 | | 0.0000 | 0.0000 |
| *Mesorhizobium* | | 0.2394 | 0.2100 | | | 0.0958 | | 0.1326 | | 0.1289 | | 0.1142 | | 0.1216 | 0.0847 |
| *Methylobacterium* | | 0.0037 | 0.0147 | | | 0.0000 | | 0.0000 | | 0.0295 | | 0.0111 | | 0.0626 | 0.0405 |
| *Methylophilaceae_uncultured* | | 0.0111 | 0.0147 | | | 0.0074 | | 0.0037 | | 0.0000 | | 0.0074 | | 0.0000 | 0.0037 |
| *Methylorosula* | | 0.0184 | 0.0111 | | | 0.0074 | | 0.0332 | | 0.0111 | | 0.0516 | | 0.0442 | 0.0221 |
| *Methylotenera* | | 0.0000 | 0.0074 | | | 0.0147 | | 0.0368 | | 0.0258 | | 0.0553 | | 0.1768 | 0.1326 |
| *Microbacterium* | | 0.0995 | 0.0221 | | | 0.0847 | | 0.0553 | | 0.0810 | | 0.0884 | | 0.1510 | 0.1437 |
| *Micrococcus* | | 0.0442 | 0.0221 | | | 0.0037 | | 0.0111 | | 0.0111 | | 0.0258 | | 0.0037 | 0.0037 |
| *Microcoleus* | | 0.0037 | 0.0000 | | | 0.0000 | | 0.0000 | | 0.0332 | | 0.0074 | | 0.0405 | 0.0553 |
| *Microgenomates_norank* | | 0.0000 | 0.0000 | | | 0.0000 | | 0.0037 | | 0.0074 | | 0.0295 | | 0.0074 | 0.0221 |
| *Microlunatus* | | 0.4678 | 0.4384 | | | 0.4126 | | 0.5010 | | 0.5599 | | 0.5489 | | 0.6815 | 0.6520 |
| *Micromonosporaceae_Unclassified* | | 0.0958 | 0.0958 | | | 0.0700 | | 0.0479 | | 0.1289 | | 0.0847 | | 0.1768 | 0.1584 |
| *Microvirga* | | 0.4089 | 0.2873 | | | 0.1915 | | 0.2210 | | 0.3389 | | 0.2726 | | 0.3905 | 0.4199 |
| *Mitochondria_norank* | | 0.0184 | 0.0258 | | | 0.0074 | | 0.0037 | | 0.0221 | | 0.0000 | | 0.0184 | 0.0074 |
| *Mizugakiibacter* | | 1.0020 | 1.1125 | | | 0.0037 | | 0.0184 | | 0.0479 | | 0.0221 | | 0.0074 | 0.0147 |
| *Moraxellaceae_uncultured* | | 0.0074 | 0.0147 | | | 0.0295 | | 0.0332 | | 0.0000 | | 0.0000 | | 0.0000 | 0.0111 |
| *Mucilaginibacter* | | 0.1289 | 0.1105 | | | 0.0000 | | 0.0000 | | 0.0111 | | 0.0111 | | 0.0111 | 0.0147 |
| *Mycobacterium* | | 0.2321 | 0.2431 | | | 0.1326 | | 0.1363 | | 0.2137 | | 0.1805 | | 0.1842 | 0.2615 |
| *Myxococcales_Unclassified* | | 0.0847 | 0.0589 | | | 0.1289 | | 0.1363 | | 0.0921 | | 0.1179 | | 0.1510 | 0.1621 |
| *Myxococcales_uncultured* | | 0.0184 | 0.0147 | | | 0.0589 | | 0.0737 | | 0.0516 | | 0.0479 | | 0.0810 | 0.1289 |
| *NKB5_norank* | | 0.0000 | 0.0221 | | | 0.0147 | | 0.0258 | | 0.0074 | | 0.0074 | | 0.0111 | 0.0074 |
| *NS11-12 marine group_norank* | | 0.0000 | 0.0147 | | | 0.0405 | | 0.0258 | | 0.0332 | | 0.0516 | | 0.0368 | 0.0479 |
| *NS72_norank* | | 0.0147 | 0.0368 | | | 0.0295 | | 0.0405 | | 0.0258 | | 0.0111 | | 0.0037 | 0.0037 |
| *NS9 marine group_norank* | | 0.0037 | 0.0221 | | | 0.0074 | | 0.0037 | | 0.0037 | | 0.0074 | | 0.0332 | 0.0147 |
| *Nannocystis* | | 0.0074 | 0.0111 | | | 0.0332 | | 0.0332 | | 0.0368 | | 0.0147 | | 0.0921 | 0.0774 |
| *Neo-b11_norank* | | 0.0589 | 0.0442 | | | 0.0626 | | 0.0332 | | 0.0700 | | 0.0958 | | 0.1068 | 0.1216 |
| *Neorhizobium* | | 0.0074 | 0.0368 | | | 0.0184 | | 0.0074 | | 0.0258 | | 0.0221 | | 0.0884 | 0.0774 |
| *Nesterenkonia* | | 0.1105 | 0.0442 | | | 0.0037 | | 0.0332 | | 0.0074 | | 0.0332 | | 0.0000 | 0.0221 |
| *Niastella* | | 0.0479 | 0.0884 | | | 0.0405 | | 0.0553 | | 0.0663 | | 0.0479 | | 0.0332 | 0.0368 |
| *Nitrolancea* | | 0.0184 | 0.0184 | | | 0.0000 | | 0.0037 | | 0.0000 | | 0.0000 | | 0.0000 | 0.0221 |
| *Nitrosomonadaceae_uncultured* | | 1.3629 | 1.0719 | | | 2.1991 | | 2.0886 | | 2.5196 | | 2.5196 | | 1.7239 | 1.7608 |
| *Nitrosospira* | | 0.2800 | 0.0958 | | | 0.2100 | | 0.1621 | | 0.1584 | | 0.1989 | | 0.2321 | 0.1842 |
| *Nitrospinaceae_uncultured* | | 0.1363 | 0.0700 | | | 0.1252 | | 0.1326 | | 0.1363 | | 0.1694 | | 0.0589 | 0.1510 |
| *Nitrospira* | | 1.0020 | 1.2709 | | | 1.7829 | | 1.5029 | | 1.9265 | | 1.8234 | | 1.0167 | 0.9062 |
| *Nocardia* | | 0.0147 | 0.0074 | | | 0.0000 | | 0.0000 | | 0.0037 | | 0.0037 | | 0.0037 | 0.0000 |
| *Nocardioides* | | 0.7588 | 0.6557 | | | 0.4310 | | 0.4126 | | 0.4862 | | 0.5525 | | 1.0462 | 1.0056 |
| *Nonomuraea* | | 0.0295 | 0.0000 | | | 0.0000 | | 0.0000 | | 0.0074 | | 0.0111 | | 0.0184 | 0.0000 |
| *Nordella* | | 0.1547 | 0.2100 | | | 0.2800 | | 0.2542 | | 0.1584 | | 0.2247 | | 0.1842 | 0.2542 |
| *Noviherbaspirillum* | | 0.3426 | 0.1289 | | | 0.5231 | | 0.5268 | | 0.6704 | | 0.6815 | | 0.8730 | 0.7220 |
| *Novosphingobium* | | 0.1142 | 0.0847 | | | 0.1363 | | 0.2063 | | 0.3536 | | 0.3021 | | 0.2394 | 0.2431 |
| *Nubsella* | | 0.0111 | 0.0037 | | | 0.0000 | | 0.0000 | | 0.0074 | | 0.0000 | | 0.0221 | 0.0295 |
| *OM190_norank* | | 0.1105 | 0.1510 | | | 0.2137 | | 0.2836 | | 0.2100 | | 0.2800 | | 0.1289 | 0.1400 |
| *OM27 clade* | | 0.0368 | 0.0626 | | | 0.1547 | | 0.1400 | | 0.1031 | | 0.0847 | | 0.0921 | 0.0921 |
| *OPB35 soil group_norank* | | 0.0553 | 0.2063 | | | 0.0700 | | 0.0295 | | 0.0516 | | 0.0368 | | 0.0258 | 0.0663 |
| *OPB56_norank* | | 0.0479 | 0.0553 | | | 0.0737 | | 0.0995 | | 0.1105 | | 0.0663 | | 0.1289 | 0.1694 |
| *Obscuribacterales_norank* | | 0.0000 | 0.0000 | | | 0.0000 | | 0.0000 | | 0.0074 | | 0.0147 | | 0.0037 | 0.0037 |
| *Ohtaekwangia* | | 0.0479 | 0.0442 | | | 0.1031 | | 0.0368 | | 0.0774 | | 0.0479 | | 0.2542 | 0.2137 |
| *Oligoflexaceae_norank* | | 0.0000 | 0.0184 | | | 0.0111 | | 0.0074 | | 0.0037 | | 0.0037 | | 0.0442 | 0.0368 |
| *Oligoflexales_norank* | | 0.0184 | 0.0074 | | | 0.0516 | | 0.0332 | | 0.0184 | | 0.0074 | | 0.0184 | 0.0295 |
| *Oligoflexus* | | 0.0000 | 0.0037 | | | 0.0037 | | 0.0074 | | 0.0111 | | 0.0184 | | 0.0147 | 0.0258 |
| *Opitutus* | | 0.1989 | 0.2984 | | | 0.2689 | | 0.2726 | | 0.3573 | | 0.2652 | | 0.5636 | 0.4568 |
| *P2-11E_norank* | | 0.2873 | 0.4199 | | | 0.3131 | | 0.3389 | | 0.4605 | | 0.4052 | | 0.0995 | 0.0884 |
| *P3OB-42_norank* | | 0.0000 | 0.0074 | | | 0.0332 | | 0.0332 | | 0.0221 | | 0.0147 | | 0.0147 | 0.0074 |
| *PAUC26f_norank* | | 0.0000 | 0.0000 | | | 0.0000 | | 0.0000 | | 0.0000 | | 0.0111 | | 0.0000 | 0.0000 |
| *PHOS-HE51_norank* | | 0.0074 | 0.0221 | | | 0.0184 | | 0.0295 | | 0.0221 | | 0.0184 | | 0.0258 | 0.0258 |
| *Paenibacillus* | | 0.2284 | 0.0995 | | | 0.0884 | | 0.0700 | | 0.2063 | | 0.1694 | | 0.0553 | 0.0810 |
| *Panacagrimonas* | | 0.0000 | 0.0000 | | | 0.0000 | | 0.0000 | | 0.0037 | | 0.0074 | | 0.0147 | 0.0295 |
| *Parafilimonas* | | 0.0442 | 0.0295 | | | 0.0147 | | 0.0368 | | 0.0589 | | 0.0295 | | 0.0147 | 0.0258 |
| *Paralcaligenes* | | 0.3647 | 0.1473 | | | 0.0000 | | 0.0000 | | 0.0000 | | 0.0000 | | 0.0000 | 0.0000 |
| *Patulibacter* | | 0.1289 | 0.1068 | | | 0.0847 | | 0.1105 | | 0.3205 | | 0.3021 | | 0.2689 | 0.2652 |
| *Pedobacter* | | 0.0000 | 0.0111 | | | 0.0000 | | 0.0000 | | 0.0000 | | 0.0000 | | 0.0000 | 0.0000 |
| *Pedomicrobium* | | 0.1216 | 0.1915 | | | 0.2026 | | 0.2100 | | 0.2063 | | 0.1768 | | 0.1473 | 0.1142 |
| *Pelagibacterium* | | 0.6815 | 0.3389 | | | 0.0184 | | 0.2284 | | 0.0184 | | 0.0368 | | 0.0258 | 0.0626 |
| *Pelosinus* | | 0.0221 | 0.0037 | | | 0.0000 | | 0.0000 | | 0.0000 | | 0.0000 | | 0.0074 | 0.0037 |
| *Phaselicystis* | | 0.0295 | 0.0295 | | | 0.0516 | | 0.0295 | | 0.1142 | | 0.0589 | | 0.0626 | 0.0589 |
| *Phenylobacterium* | | 1.0130 | 0.9799 | | | 0.4789 | | 0.5010 | | 0.5194 | | 0.5047 | | 0.5452 | 0.7441 |
| *Phycisphaeraceae_uncultured* | | 0.0147 | 0.0000 | | | 0.0258 | | 0.0111 | | 0.0147 | | 0.0074 | | 0.0000 | 0.0111 |
| *Phyllobacteriaceae_Unclassified* | | 0.5157 | 0.3242 | | | 0.1179 | | 0.1768 | | 0.1179 | | 0.0958 | | 0.0553 | 0.1400 |
| *Phyllobacterium* | | 0.0516 | 0.0516 | | | 0.0442 | | 0.0147 | | 0.0442 | | 0.0368 | | 0.0184 | 0.0295 |
| *Pir4 lineage* | | 0.0442 | 0.0884 | | | 0.0368 | | 0.0442 | | 0.0626 | | 0.0258 | | 0.0368 | 0.0332 |
| *Pirellula* | | 0.1216 | 0.2652 | | | 0.3315 | | 0.3573 | | 0.4163 | | 0.5231 | | 0.3278 | 0.3426 |
| *Piscinibacter* | | 0.6631 | 0.4752 | | | 1.0425 | | 0.9651 | | 1.0719 | | 1.2156 | | 1.2856 | 1.3224 |
| *Pla1 lineage_norank* | | 0.0074 | 0.0111 | | | 0.0074 | | 0.0184 | | 0.0074 | | 0.0074 | | 0.0000 | 0.0037 |
| *Pla3 lineage_norank* | | 0.0000 | 0.0000 | | | 0.0037 | | 0.0000 | | 0.0111 | | 0.0000 | | 0.0000 | 0.0000 |
| *Pla4 lineage_norank* | | 0.0258 | 0.0589 | | | 0.0221 | | 0.0405 | | 0.0479 | | 0.0405 | | 0.0553 | 0.0221 |
| *Planctomyces* | | 0.1363 | 0.1105 | | | 0.1179 | | 0.1326 | | 0.1473 | | 0.0884 | | 0.1400 | 0.0884 |
| *Planctomycetaceae_Unclassified* | | 0.0737 | 0.1142 | | | 0.1842 | | 0.1805 | | 0.2652 | | 0.2505 | | 0.2026 | 0.2137 |
| *Planctomycetaceae_uncultured* | | 0.3610 | 0.2947 | | | 0.2873 | | 0.3463 | | 0.4752 | | 0.4384 | | 0.3389 | 0.3868 |
| *Planctomycetes_Unclassified* | | 0.0147 | 0.0147 | | | 0.0037 | | 0.0000 | | 0.0111 | | 0.0037 | | 0.0000 | 0.0000 |
| *Planococcaceae_Unclassified* | | 0.1326 | 0.1584 | | | 0.0737 | | 0.0810 | | 0.1289 | | 0.1289 | | 0.0553 | 0.0663 |
| *Planosporangium* | | 0.0332 | 0.0258 | | | 0.0147 | | 0.0221 | | 0.0295 | | 0.0258 | | 0.0147 | 0.0074 |
| *Plantactinospora* | | 0.2800 | 0.3021 | | | 0.2284 | | 0.2063 | | 0.2947 | | 0.2358 | | 0.4826 | 0.5341 |
| *Polaromonas* | | 0.0847 | 0.0332 | | | 0.0074 | | 0.0111 | | 0.0147 | | 0.0258 | | 0.1989 | 0.2137 |
| *Polyangiaceae_Unclassified* | | 0.0000 | 0.0000 | | | 0.0111 | | 0.0258 | | 0.0332 | | 0.0037 | | 0.0921 | 0.0553 |
| *Polyangiaceae_uncultured* | | 0.0258 | 0.0147 | | | 0.0332 | | 0.0295 | | 0.0258 | | 0.0332 | | 0.0626 | 0.1216 |
| *Polyangium* | | 0.0147 | 0.0111 | | | 0.0368 | | 0.0147 | | 0.0368 | | 0.0147 | | 0.0553 | 0.0774 |
| *Polycyclovorans* | | 0.0000 | 0.0000 | | | 0.0037 | | 0.0000 | | 0.0074 | | 0.0221 | | 0.0111 | 0.0037 |
| *Promicromonospora* | | 0.0000 | 0.0111 | | | 0.0037 | | 0.0037 | | 0.0258 | | 0.0258 | | 0.0037 | 0.0074 |
| *Propionibacterium* | | 0.0295 | 0.0000 | | | 0.0000 | | 0.0037 | | 0.0037 | | 0.0000 | | 0.0000 | 0.0000 |
| *Proteobacteria_Unclassified* | | 0.0000 | 0.0000 | | | 0.0479 | | 0.0074 | | 0.0147 | | 0.0000 | | 0.0000 | 0.0037 |
| *Pseudenhygromyxa* | | 0.0405 | 0.0258 | | | 0.0184 | | 0.0074 | | 0.0184 | | 0.0037 | | 0.0074 | 0.0295 |
| *Pseudoclavibacter* | | 0.0442 | 0.0516 | | | 0.0184 | | 0.0074 | | 0.0074 | | 0.0037 | | 0.0184 | 0.0221 |
| *Pseudoduganella* | | 0.0368 | 0.0258 | | | 0.0295 | | 0.0295 | | 0.0921 | | 0.0884 | | 0.0589 | 0.0921 |
| *Pseudolabrys* | | 0.1363 | 0.1510 | | | 0.2210 | | 0.2063 | | 0.2063 | | 0.1805 | | 0.1437 | 0.1252 |
| *Pseudomonas* | | 0.1142 | 0.1289 | | | 0.1437 | | 0.1400 | | 0.1437 | | 0.1805 | | 0.1731 | 0.1805 |
| *Pseudonocardia* | | 0.3315 | 0.4236 | | | 0.3499 | | 0.5010 | | 0.3573 | | 0.3610 | | 0.7588 | 0.6925 |
| *Pseudospirillum* | | 0.0037 | 0.0000 | | | 0.0037 | | 0.0074 | | 0.0037 | | 0.0111 | | 0.0921 | 0.0847 |
| *Pseudoxanthomonas* | | 0.1879 | 0.2431 | | | 0.1510 | | 0.0810 | | 0.0884 | | 0.0847 | | 0.1510 | 0.1400 |
| *RB41_norank* | | 3.5142 | 1.8455 | | | 6.4059 | | 5.7723 | | 5.7760 | | 5.8349 | | 3.6800 | 3.4995 |
| *Ramlibacter* | | 0.4494 | 0.1842 | | | 0.4310 | | 0.4347 | | 0.6299 | | 0.6962 | | 0.5673 | 0.5857 |
| *Reyranella* | | 0.7294 | 0.8178 | | | 0.8878 | | 0.6741 | | 1.0093 | | 0.7883 | | 0.5268 | 0.5194 |
| *Rhizobiales_Unclassified* | | 0.4936 | 0.3463 | | | 0.6004 | | 0.5083 | | 0.5083 | | 0.5231 | | 0.4052 | 0.4236 |
| *Rhizobium* | | 0.1068 | 0.0884 | | | 0.0368 | | 0.0332 | | 0.0700 | | 0.0589 | | 0.0921 | 0.0810 |
| *Rhizocola* | | 0.0810 | 0.1068 | | | 0.0737 | | 0.0700 | | 0.0626 | | 0.0958 | | 0.0405 | 0.0479 |
| *Rhizomicrobium* | | 0.2910 | 0.6262 | | | 0.0516 | | 0.0589 | | 0.0810 | | 0.0810 | | 0.2358 | 0.2615 |
| *Rhodanobacter* | | 7.2936 | 7.9235 | | | 0.0884 | | 0.1289 | | 0.1068 | | 0.0995 | | 0.0700 | 0.0737 |
| *Rhodobiaceae_uncultured* | | 0.1768 | 0.1694 | | | 0.1289 | | 0.1768 | | 0.1842 | | 0.1658 | | 0.1731 | 0.1805 |
| *Rhodococcus* | | 0.0037 | 0.0074 | | | 0.0074 | | 0.0037 | | 0.0258 | | 0.0258 | | 0.0516 | 0.0737 |
| *Rhodocytophaga* | | 0.0147 | 0.0221 | | | 0.0147 | | 0.0368 | | 0.0332 | | 0.0442 | | 0.0442 | 0.0332 |
| *Rhodoplanes* | | 0.1989 | 0.1915 | | | 0.4752 | | 0.2984 | | 0.3647 | | 0.3573 | | 0.3205 | 0.2873 |
| *Rhodopseudomonas* | | 0.0847 | 0.0958 | | | 0.0405 | | 0.0405 | | 0.0368 | | 0.0258 | | 0.1216 | 0.0995 |
| *Rhodospirillaceae_Unclassified* | | 0.0184 | 0.0221 | | | 0.0111 | | 0.0184 | | 0.0184 | | 0.0074 | | 0.0074 | 0.0074 |
| *Rhodospirillaceae_uncultured* | | 0.6262 | 0.6336 | | | 0.3720 | | 0.3868 | | 0.4236 | | 0.3905 | | 0.2800 | 0.2615 |
| *Rhodospirillales_Unclassified* | | 0.0368 | 0.0663 | | | 0.0147 | | 0.0000 | | 0.0074 | | 0.0037 | | 0.0037 | 0.0221 |
| *Rhodovarius* | | 0.0074 | 0.0074 | | | 0.0000 | | 0.0000 | | 0.0074 | | 0.0074 | | 0.0074 | 0.0037 |
| *Rhodovastum* | | 0.3315 | 0.2210 | | | 0.0000 | | 0.0000 | | 0.0000 | | 0.0000 | | 0.0000 | 0.0000 |
| *Rikenellaceae RC9 gut group* | | 0.0332 | 0.0000 | | | 0.0000 | | 0.0000 | | 0.0000 | | 0.0000 | | 0.0000 | 0.0000 |
| *Romboutsia* | | 0.0000 | 0.0037 | | | 0.0037 | | 0.0111 | | 0.0000 | | 0.0000 | | 0.0000 | 0.0037 |
| *Roseiflexus* | | 0.9467 | 1.0204 | | | 1.2046 | | 1.0683 | | 0.9872 | | 1.0977 | | 1.0056 | 0.9799 |
| *Roseomonas* | | 0.0037 | 0.0037 | | | 0.0111 | | 0.0111 | | 0.0184 | | 0.0074 | | 0.0221 | 0.0479 |
| *Rubellimicrobium* | | 0.0037 | 0.0037 | | | 0.0000 | | 0.0037 | | 0.0147 | | 0.0221 | | 0.0553 | 0.0958 |
| *Rubrobacter* | | 0.0626 | 0.2615 | | | 0.0700 | | 0.0405 | | 0.0737 | | 0.0810 | | 0.0663 | 0.1400 |
| *Ruminococcaceae_uncultured* | | 0.0000 | 0.0074 | | | 0.0000 | | 0.0000 | | 0.0000 | | 0.0000 | | 0.0000 | 0.0000 |
| *Rummeliibacillus* | | 0.0037 | 0.0074 | | | 0.0000 | | 0.0000 | | 0.0000 | | 0.0000 | | 0.0000 | 0.0000 |
| *S0134 terrestrial group_norank* | | 0.0626 | 0.0774 | | | 0.0958 | | 0.0884 | | 0.1400 | | 0.1289 | | 0.1768 | 0.1805 |
| *S085_norank* | | 0.0405 | 0.0295 | | | 0.0295 | | 0.0884 | | 0.0442 | | 0.0442 | | 0.0111 | 0.0774 |
| *SC-I-84_norank* | | 3.3853 | 1.9892 | | | 4.9103 | | 4.7372 | | 4.1146 | | 4.5088 | | 3.2895 | 3.0022 |
| *SHA-109_norank* | | 0.0184 | 0.0074 | | | 0.0074 | | 0.0111 | | 0.0037 | | 0.0074 | | 0.0147 | 0.0037 |
| *SHA-26_norank* | | 0.0000 | 0.0074 | | | 0.0111 | | 0.0147 | | 0.0111 | | 0.0221 | | 0.0111 | 0.0074 |
| *SJA-149_norank* | | 0.0184 | 0.0037 | | | 0.0074 | | 0.0111 | | 0.0184 | | 0.0405 | | 0.0184 | 0.0295 |
| *SJA-28_norank* | | 0.0184 | 0.0147 | | | 0.0147 | | 0.0037 | | 0.0221 | | 0.0037 | | 0.0074 | 0.0074 |
| *SM1A02* | | 0.0184 | 0.0295 | | | 0.0184 | | 0.0111 | | 0.0147 | | 0.0221 | | 0.0258 | 0.0147 |
| *SM2D12_norank* | | 0.0000 | 0.0000 | | | 0.0000 | | 0.0147 | | 0.0000 | | 0.0000 | | 0.0000 | 0.0000 |
| *SM2F11_norank* | | 0.0037 | 0.0000 | | | 0.0000 | | 0.0111 | | 0.0000 | | 0.0000 | | 0.0000 | 0.0000 |
| *Saccharibacteria_norank* | | 0.0000 | 0.0037 | | | 0.0368 | | 0.0737 | | 0.0074 | | 0.0184 | | 0.0111 | 0.0037 |
| *Salinisphaeraceae_uncultured* | | 0.0147 | 0.0111 | | | 0.0368 | | 0.0111 | | 0.0147 | | 0.0368 | | 0.0111 | 0.0111 |
| *Sandaracinaceae_uncultured* | | 0.0405 | 0.0553 | | | 0.0921 | | 0.0995 | | 0.0884 | | 0.0626 | | 0.2505 | 0.1879 |
| *Sandaracinus* | | 0.0037 | 0.0221 | | | 0.0074 | | 0.0037 | | 0.0221 | | 0.0147 | | 0.0111 | 0.0000 |
| *Saprospiraceae_uncultured* | | 0.0221 | 0.0810 | | | 0.1216 | | 0.1068 | | 0.0921 | | 0.0553 | | 0.2321 | 0.1915 |
| *Schlesneria* | | 0.0000 | 0.0000 | | | 0.0000 | | 0.0000 | | 0.0037 | | 0.0037 | | 0.0000 | 0.0111 |
| *Segetibacter* | | 0.0442 | 0.0037 | | | 0.0442 | | 0.0184 | | 0.0810 | | 0.0589 | | 0.2137 | 0.1805 |
| *Sh765B-TzT-29_norank* | | 0.0147 | 0.0295 | | | 0.0700 | | 0.0847 | | 0.0516 | | 0.0626 | | 0.0295 | 0.0332 |
| *Singulisphaera* | | 0.1142 | 0.1289 | | | 0.0516 | | 0.0626 | | 0.0368 | | 0.0921 | | 0.0479 | 0.0589 |
| *Skermanella* | | 0.3573 | 0.4163 | | | 0.3794 | | 0.3868 | | 0.4236 | | 0.3868 | | 0.7699 | 0.9025 |
| *Solimonadaceae_Unclassified* | | 0.0000 | 0.0000 | | | 0.0037 | | 0.0037 | | 0.0037 | | 0.0037 | | 0.0037 | 0.0037 |
| *Solirubrobacter* | | 0.3168 | 0.3205 | | | 0.4236 | | 0.3720 | | 0.3242 | | 0.3905 | | 0.6446 | 0.6594 |
| *Solirubrobacterales_Unclassified* | | 0.0405 | 0.0553 | | | 0.0479 | | 0.0589 | | 0.0847 | | 0.0553 | | 0.1252 | 0.0774 |
| *Sorangium* | | 0.1216 | 0.0737 | | | 0.0663 | | 0.0774 | | 0.0847 | | 0.0774 | | 0.1547 | 0.1842 |
| *Sphingobacteriaceae_Unclassified* | | 0.0405 | 0.0589 | | | 0.0516 | | 0.0553 | | 0.0737 | | 0.0589 | | 0.0884 | 0.0847 |
| *Sphingobium* | | 0.0184 | 0.0147 | | | 0.0037 | | 0.0111 | | 0.0000 | | 0.0037 | | 0.0037 | 0.0000 |
| *Sphingomonadaceae_Unclassified* | | 0.0184 | 0.0258 | | | 0.0000 | | 0.0000 | | 0.0000 | | 0.0000 | | 0.0000 | 0.0000 |
| *Sphingomonadales_Unclassified* | | 0.0074 | 0.0553 | | | 0.0184 | | 0.0147 | | 0.0111 | | 0.0111 | | 0.0368 | 0.0368 |
| *Sphingomonas* | | 0.7994 | 2.6743 | | | 0.6373 | | 0.4826 | | 0.4457 | | 0.2321 | | 0.4715 | 0.6446 |
| *Sphingopyxis* | | 0.0000 | 0.0037 | | | 0.0000 | | 0.0000 | | 0.0037 | | 0.0074 | | 0.0184 | 0.0184 |
| *Sporichthyaceae_uncultured* | | 0.0921 | 0.1031 | | | 0.1216 | | 0.1068 | | 0.1142 | | 0.0516 | | 0.1142 | 0.1621 |
| *Steroidobacter* | | 0.6041 | 0.2652 | | | 0.5820 | | 0.5378 | | 0.2615 | | 0.3499 | | 0.3610 | 0.2800 |
| *Streptomyces* | | 0.6004 | 0.4310 | | | 0.3794 | | 0.3389 | | 0.5341 | | 0.6299 | | 0.5194 | 0.5231 |
| *Streptosporangiaceae_Unclassified* | | 0.0111 | 0.0221 | | | 0.0037 | | 0.0074 | | 0.0074 | | 0.0111 | | 0.0111 | 0.0147 |
| *Streptosporangium* | | 0.0147 | 0.0332 | | | 0.0147 | | 0.0111 | | 0.0405 | | 0.0074 | | 0.0405 | 0.0221 |
| *Subgroup 11_norank* | | 0.0589 | 0.0884 | | | 0.0663 | | 0.0958 | | 0.0884 | | 0.0884 | | 0.0700 | 0.0737 |
| *Subgroup 13_norank* | | 0.0037 | 0.0000 | | | 0.0000 | | 0.0037 | | 0.0037 | | 0.0221 | | 0.0000 | 0.0000 |
| *Subgroup 15_norank* | | 0.0000 | 0.0147 | | | 0.0258 | | 0.0147 | | 0.0184 | | 0.0184 | | 0.0111 | 0.0258 |
| *Subgroup 17_norank* | | 0.2394 | 0.2100 | | | 0.2063 | | 0.2284 | | 0.2615 | | 0.2394 | | 0.0884 | 0.0958 |
| *Subgroup 18_norank* | | 0.0258 | 0.0221 | | | 0.0553 | | 0.0221 | | 0.0516 | | 0.0553 | | 0.0111 | 0.0184 |
| *Subgroup 25_norank* | | 0.0147 | 0.0589 | | | 0.0626 | | 0.0663 | | 0.0921 | | 0.0700 | | 0.0184 | 0.0147 |
| *Subgroup 2_norank* | | 0.0258 | 0.0295 | | | 0.0368 | | 0.0184 | | 0.0184 | | 0.0074 | | 0.0147 | 0.0037 |
| *Subgroup 5_norank* | | 0.3278 | 0.4494 | | | 0.5231 | | 0.4199 | | 0.5194 | | 0.5783 | | 0.3021 | 0.3242 |
| *Subgroup 6_norank* | | 7.1315 | 7.6473 | | | 10.0564 | | 9.7248 | | 9.4596 | | 10.0674 | | 8.9697 | 8.7266 |
| *Subgroup 7_norank* | | 0.3057 | 0.4715 | | | 0.7146 | | 0.8362 | | 0.8693 | | 0.9099 | | 0.9577 | 0.8914 |
| *SubsectionIV_FamilyI_norank* | | 0.0000 | 0.0074 | | | 0.0037 | | 0.0037 | | 0.0074 | | 0.0111 | | 0.0074 | 0.0000 |
| *Sva0725_norank* | | 0.0589 | 0.0663 | | | 0.1031 | | 0.0553 | | 0.1179 | | 0.1142 | | 0.0368 | 0.0442 |
| *Syntrophaceae_uncultured* | | 0.0000 | 0.0000 | | | 0.0037 | | 0.0111 | | 0.0000 | | 0.0000 | | 0.0000 | 0.0037 |
| *TK10_norank* | | 1.0940 | 0.8436 | | | 1.1567 | | 1.2340 | | 1.3703 | | 1.4072 | | 0.7257 | 0.8178 |
| *TK34_norank* | | 0.0037 | 0.0037 | | | 0.0147 | | 0.0000 | | 0.0221 | | 0.0184 | | 0.0111 | 0.0184 |
| *TM6_norank* | | 0.0111 | 0.0000 | | | 0.0258 | | 0.0037 | | 0.0111 | | 0.0074 | | 0.0000 | 0.0000 |
| *TRA3-20_norank* | | 0.3684 | 0.2321 | | | 0.7220 | | 0.4052 | | 0.5231 | | 0.5857 | | 0.5710 | 0.5968 |
| *Taibaiella* | | 0.0295 | 0.0221 | | | 0.0000 | | 0.0000 | | 0.0000 | | 0.0000 | | 0.0000 | 0.0000 |
| *Terriglobus* | | 0.0111 | 0.0037 | | | 0.0000 | | 0.0000 | | 0.0000 | | 0.0111 | | 0.0000 | 0.0000 |
| *Thermosporotrichaceae_uncultured* | | 0.0111 | 0.0111 | | | 0.0000 | | 0.0000 | | 0.0000 | | 0.0074 | | 0.0000 | 0.0074 |
| *Tumebacillus* | | 0.0442 | 0.0774 | | | 0.0147 | | 0.0184 | | 0.0368 | | 0.0368 | | 0.0258 | 0.0111 |
| *Umezawaea* | | 0.0074 | 0.0000 | | | 0.0000 | | 0.0111 | | 0.0000 | | 0.0037 | | 0.0737 | 0.1031 |
| *Vampirovibrionales_norank* | | 0.0000 | 0.0037 | | | 0.0037 | | 0.0000 | | 0.0000 | | 0.0000 | | 0.0000 | 0.0000 |
| *Variibacter* | | 0.4273 | 0.4310 | | | 0.7883 | | 0.8288 | | 0.6852 | | 0.8509 | | 0.5894 | 0.5746 |
| *Verrucomicrobia_Unclassified* | | 0.0000 | 0.0074 | | | 0.0000 | | 0.0000 | | 0.0000 | | 0.0000 | | 0.0000 | 0.0000 |
| *Virgisporangium* | | 0.1068 | 0.0258 | | | 0.0479 | | 0.0479 | | 0.0810 | | 0.0700 | | 0.0810 | 0.0700 |
| *Vulgatibacter* | | 0.0111 | 0.0147 | | | 0.0037 | | 0.0000 | | 0.0074 | | 0.0000 | | 0.0000 | 0.0074 |
| *WCHB1-60_norank* | | 0.0147 | 0.0258 | | | 0.0111 | | 0.0074 | | 0.0147 | | 0.0332 | | 0.0479 | 0.0442 |
| *WD2101 soil group_norank* | | 1.7129 | 1.9560 | | | 2.9985 | | 2.9322 | | 2.7922 | | 2.5859 | | 2.7259 | 2.3354 |
| *Xanthobacteraceae_Unclassified* | | 0.2800 | 0.3094 | | | 0.4568 | | 0.3831 | | 0.4899 | | 0.4789 | | 0.3573 | 0.3389 |
| *Xanthobacteraceae_uncultured* | | 0.0037 | 0.0258 | | | 0.0405 | | 0.0663 | | 0.0516 | | 0.0405 | | 0.0589 | 0.0589 |
| *Xanthomonadaceae_Unclassified* | | 0.7957 | 1.0020 | | | 0.8583 | | 0.9099 | | 0.4899 | | 0.4420 | | 0.4973 | 0.5378 |
| *Xanthomonadaceae_uncultured* | | 0.0221 | 0.0332 | | | 0.0184 | | 0.0295 | | 0.0295 | | 0.0479 | | 0.0810 | 0.1437 |
| *Xanthomonadales Incertae Sedis_uncultured* | | 0.3168 | 0.1768 | | | 0.5341 | | 0.5304 | | 0.3610 | | 0.2542 | | 0.1915 | 0.1547 |
| *Xanthomonadales_uncultured* | | 0.3278 | 0.2358 | | | 0.3131 | | 0.3868 | | 0.2321 | | 0.2468 | | 0.2873 | 0.3205 |
| *YNPFFP1_norank* | | 0.0589 | 0.0147 | | | 0.0221 | | 0.0184 | | 0.0184 | | 0.0111 | | 0.0258 | 0.0368 |
| *Zavarzinella* | | 0.0295 | 0.0368 | | | 0.0368 | | 0.0405 | | 0.0626 | | 0.0958 | | 0.0516 | 0.0516 |
| *cvE6_norank* | | 0.0000 | 0.0111 | | | 0.0000 | | 0.0000 | | 0.0000 | | 0.0000 | | 0.0000 | 0.0000 |
| *env.OPS 17_norank* | | 0.0221 | 0.0258 | | | 0.0589 | | 0.0516 | | 0.0405 | | 0.0405 | | 0.0995 | 0.1142 |
| *mle1-27_norank* | | 0.0332 | 0.0442 | | | 0.0847 | | 0.0958 | | 0.0368 | | 0.0442 | | 0.0553 | 0.0295 |
| *vadinHA49_norank* | | 0.0000 | 0.0111 | | | 0.0147 | | 0.0037 | | 0.0074 | | 0.0074 | | 0.0479 | 0.0295 |


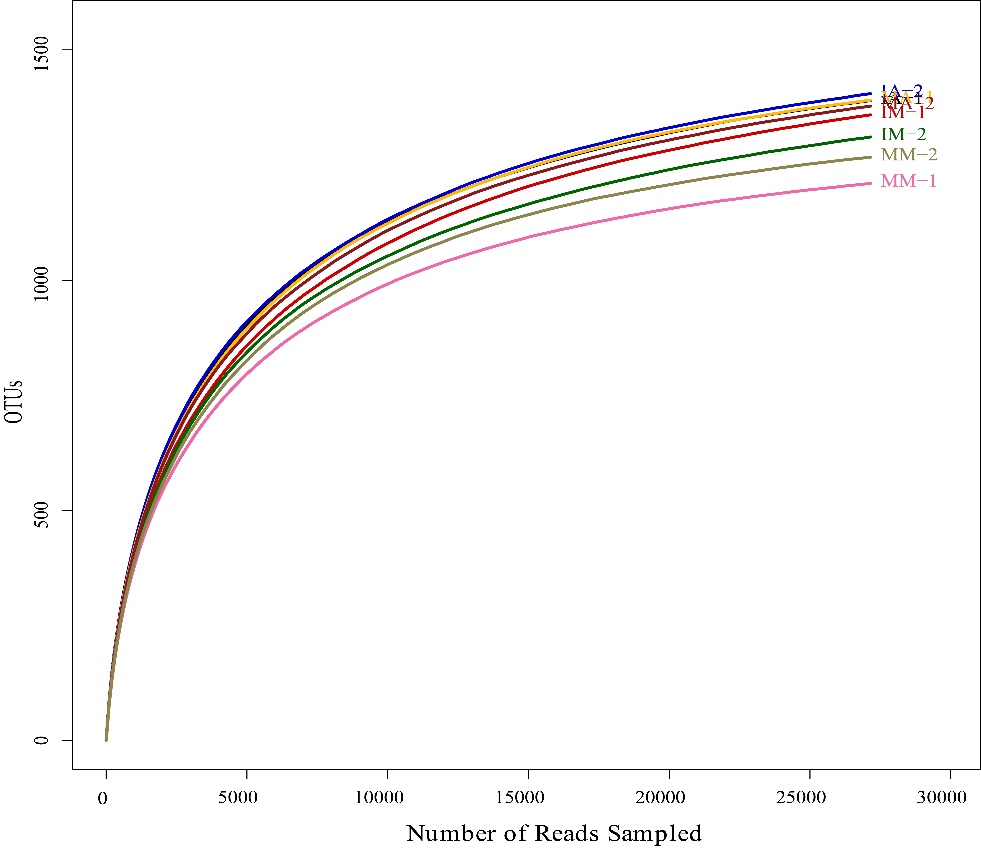


Fig. S1 Rarefaction curves of the different treatments at 3% dissimilarity levels.

Note：MM-1 and MM-2 represent two repeats of MM, IM-1 and IM-2 represent two repeats of IM, MA-1 and MA-2 represent two repeats of MA, and IA-1 and IA-2 represent two repeats of IA. The same abbreviations appear below.
